# Activin Receptor Ligand Blocking and Cancer Have Distinct Effects on Protein and Redox Homeostasis in Skeletal Muscle and Liver

**DOI:** 10.3389/fphys.2018.01917

**Published:** 2019-01-18

**Authors:** Jaakko Hentilä, Tuuli A. Nissinen, Ayhan Korkmaz, Sanna Lensu, Mika Silvennoinen, Arja Pasternack, Olli Ritvos, Mustafa Atalay, Juha J. Hulmi

**Affiliations:** ^1^Faculty of Sport and Health Sciences, Neuromuscular Research Center, University of Jyväskylä, Jyväskylä, Finland; ^2^Institute of Biomedicine, Physiology, University of Eastern Finland, Kuopio, Finland; ^3^Department of Physiology, Faculty of Medicine, University of Helsinki, Helsinki, Finland

**Keywords:** cancer cachexia, autophagy, myostatin, activin, unfolded protein response, glutathione, oxidative stress/redox

## Abstract

Muscle wasting in cancer cachexia can be alleviated by blocking activin receptor type 2 (ACVR2) ligands through changes in protein synthesis/degradation. These changes in cellular and protein metabolism may alter protein homeostasis. First, we elucidated the acute (1–2 days) and 2-week effects of blocking ACVR2 ligands by soluble activin receptor 2B (sACVR2B-Fc) on unfolded protein response (UPR), heat shock proteins (HSPs) and redox balance in a healthy mouse skeletal muscle. Second, we examined UPR, autophagy and redox balance with or without sACVR2B-Fc administration in muscle and liver of C26 tumor-bearing mice. The indicators of UPR and HSPs were not altered 1–2 days after a single sACVR2B-Fc administration in healthy muscles, but protein carbonyls increased (*p* < 0.05). Two weeks of sACVR2B-Fc administration increased muscle size, which was accompanied by increased UPR markers: GRP78 (*p* < 0.05), phosphorylated eIF2α (*p* < 0.01) and HSP47 (*p* < 0.01). Additionally, protein carbonyls and reduced form of glutathione increased (GSH) (*p* < 0.05). On the other hand, C26 cancer cachexia manifested decreased UPR markers (p-eIF2α, HSP47, p-JNK; *p* < 0.05) and antioxidant GSH (*p* < 0.001) in muscle, whereas the ratio of oxidized to reduced glutathione increased (GSSG/GSH; *p* < 0.001). Administration of sACVR2B-Fc prevented the decline in GSH and increased some of the UPR indicators in tumor-bearing mice. Additionally, autophagy markers LC3II/I (*p* < 0.05), Beclin-1 (*p* < 0.01), and P62 (*p* < 0.05) increased in the skeletal muscle of tumor-bearing mice. Finally, indicators of UPR, PERK, p-eIF2α and GRP78, increased (*p* < 0.05), whereas ATF4 was strongly decreased (*p* < 0.01) in the liver of tumor-bearing mice while sACVR2B-Fc had no effect. Muscle GSH and many of the altered UPR indicators correlated with tumor mass, fat mass and body mass loss. In conclusion, experimental cancer cachexia is accompanied by distinct and tissue-specific changes in proteostasis. Muscle hypertrophy induced by blocking ACVR2B ligands may be accompanied by the induction of UPR and increased protein carbonyls but blocking ACVR2B ligands may upregulate antioxidant protection.

## Introduction

Cachexia is characterized by loss of skeletal muscle mass with or without reduction of fat mass and is common in diseases such as cancer, chronic obstructive pulmonary disease and sepsis (Fearon et al., 2011; Argiles et al., 2014; von Haehling and Anker, 2014). Loss of skeletal muscle mass is an independent predictor of bad prognosis in cancer (Martin et al., 2013; Choi et al., 2015). In preclinical animal models, prevention of cancer-induced muscle loss without an effect on tumor growth (Cai et al., 2004; Zhou et al., 2010; Nissinen et al., 2018) suggests possible causality between maintenance of muscle mass and improved survival in cachexia, but the underlying mechanisms are unknown.

Cellular protein homeostasis (i.e., proteostasis) is maintained through several integrated biological processes. For instance, undesirably modified or misfolded proteins are degraded by ubiquitin proteasome or autophagy lysosome pathways (Schneider and Bertolotti, 2015; Sandri, 2016). Additionally, disrupted cellular homeostasis induced by, for example, robustly increased protein synthesis, aberrant redox control or unbalanced endoplasmic reticulum (ER) calcium homeostasis can lead to accumulation of misfolded proteins into the lumen of ER, a process also known as ER stress (Cao and Kaufman, 2014; Hetz et al., 2015). Cells respond to ER stress in order to maintain homeostasis by activating unfolded protein response (UPR). It is a process which restores ER homeostasis by various mechanisms, such as increasing protein folding machinery, degrading misfolded proteins, suppressing protein synthesis and inducing autophagy. If ER stress is not rescued by UPR, metabolic impairments or apoptosis may occur (Hetz et al., 2015).

In skeletal muscle, disrupted protein homeostasis has been observed in wasting conditions and muscular dystrophies. This has been manifested as increased ER stress and oxidative stress in muscular dystrophies (Renjini et al., 2012; Screen et al., 2014; Hulmi et al., 2016), and there is some evidence that ER and oxidative stress are increased in some experimental cancer cachexia models as well (Der-Torossian et al., 2013; Ham et al., 2014; Bohnert et al., 2016). In skeletal muscle, cancer cachexia has been reported to induce ER stress and UPR in 2 experimental animal models: Lewis lung carcinoma (LLC) and cachectic Apc^min/+^ mice (Bohnert et al., 2016). Interestingly, UPR inhibition in cancer cachexia by chemical chaperone 4-PBA accelerated muscle wasting in LLC and Apc^min/+^ mice (Bohnert et al., 2016). Very recently, skeletal muscle specific ablation of PERK induced muscle wasting in healthy mice and further increased muscle wasting in LLC tumor bearing mice (Gallot et al., 2018). In addition to skeletal muscle, increased ER stress in the liver has been observed in cachectic Apc^min/+^ mice (Narsale et al., 2015). Upregulated and/or impaired autophagy has also been reported in cancer cachexia (Penna et al., 2013; Aversa et al., 2016). It is unknown if UPR is ubiquitously induced in other wasting and cachexia models such as in C26 tumor-bearing mice.

Blocking activin receptor ligands using the soluble ligand binding domain of a type 2B activin receptor fused to the Fc domain (sACVR2B-Fc) rapidly increases muscle size in mice (Lee et al., 2005; Zhou et al., 2010; Pistilli et al., 2011; Hoogaars et al., 2012; Hulmi et al., 2013a) and in humans (Attie et al., 2013). This occurs through increased protein synthesis (Hulmi et al., 2013a; Nissinen et al., 2016) but possibly in some situations through decreased protein degradation (Zhou et al., 2010). Increased muscle mass may not, however, always translate into better muscle function (Amthor et al., 2007; Relizani et al., 2014), perhaps in part due to qualitative changes in muscle (Amthor et al., 2007; Relizani et al., 2014; Hulmi et al., 2016; Marabita et al., 2016). However, the effect of rapid muscle hypertrophy induced by an activin receptor ligand blockade on protein homeostasis is currently unknown. We previously reported improved survival in C26 tumor-bearing mice with sACVR2B-Fc treatment (Nissinen et al., 2018). Cachectic mice had decreased protein synthesis in skeletal muscle and increased protein synthesis in the liver, the latter of which was alleviated by sACVR2B-Fc treatment (Nissinen et al., 2018). Thus, we further investigated the effects of cancer cachexia and sACVR2B-Fc treatment on protein homeostasis in muscle and the liver.

The purpose of this study was to elucidate the effects of muscle wasting induced by cancer cachexia, and muscle hypertrophy induced by sACVR2B-Fc treatment on biological processes contributing to the protein and redox homeostasis in skeletal muscle. Furthermore, because skeletal muscle and the liver are known to crosstalk (Whitham et al., 2018), we explored the effects of cancer and sACVR2B-Fc treatment on protein homeostasis in the liver as well. We hypothesized that both rapid atrophy and hypertrophy would alter several integrated processes that regulate protein homeostasis.

## Materials and Methods

### Ethics Statement

The treatment of the animals was in strict accordance with the European convention for the protection of vertebrate animals used for experimental and other scientific purposes. The protocols were approved by the National Animal Experiment Board (Permit No.: ESLH-2009-08528/Ym-23 and ESAVI/10137/04.10.07/2014).

### Animals and Cells

#### Single and 2-Week Administration of sACVR2B-Fc on Healthy Mice

Male, 6–7-week-old C57Bl/10SnJ mice were used as previously described (Hulmi et al., 2013a). The mice were purchased from the Jackson Laboratory (Bar Harbor, ME, United States).

#### The Cancer Cachexia Experiments

In the cancer cachexia experiments, 5–6-week-old male BALB/*c* (BALB/cAnCrl) mice (Charles River Laboratories) were used. All the mice were housed in standard conditions (temperature 22°C, light from 8:00 AM to 8:00 PM) and had free access to tap water and food pellets (R36, 4% fat, 55.7% carbohydrate, 18.5% protein, 3 kcal/g, Labfor, Stockholm, Sweden).

#### Tumor Cell Culture

Complete Dulbecco’s Modified Eagle’s Medium (DMEM, high glucose, GlutaMAX^TM^ Supplement pyruvate, Gibco^TM^, Life Technologies) supplemented with penicillin (100 U/ml), streptomycin (100 μg/ml) and 10% FBS was used for the maintenance of the colon 26 carcinoma cells as previously described (Nissinen et al., 2018).

### Experimental Design

**FIGURE 1:**
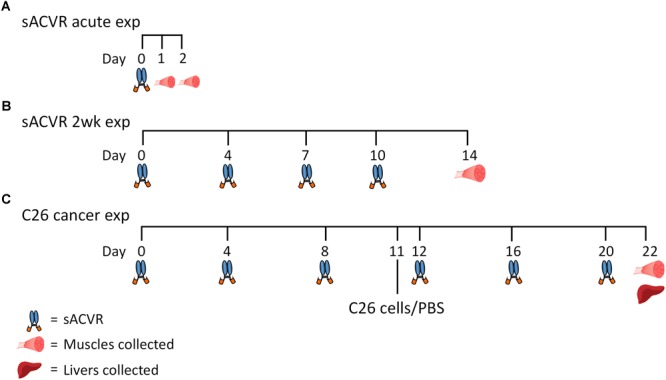
Overall study design. **(A)** Healthy mice were administered (i.p.) with a single dose of vehicle (PBS) or soluble activin receptor type 2B (sACVR) to block ACVR2B ligands. Muscles were collected either 1 or 2 days after the single sACVR or vehicle administration (*n*= 6–7 per group). **(B)** Healthy mice were administered (i.p.) with vehicle (PBS) or sACVR for 2 weeks (1–2 times per week) and muscles were collected (*n* = 6 in PBS and *n* = 11 in sACVR 2wk). **(C)** C26 cancer experiment: (1) CTRL (+PBS, *n* = 9), (2) C26 + PBS (*n* = 7), (3) C26 + sACVR/b (*n* = 7), and (4) C26 + sACVR/c (*n* = 8). Cancer groups were inoculated subcutaneously (s.c.) with C26 cells on day 11. The vehicle (PBS) and sACVR2B-Fc were administered (i.p.) every fourth day. Muscles and livers were collected 11 days after the C26 cell inoculation.

This study consisted of three separate experiments (Figure 1), which are described in detail below.

#### Acute sACVR2B-Fc Experiment in Healthy Mice

To study the acute effects of sACVR2B-Fc administration in healthy skeletal muscle, mice were divided into three groups that were euthanized 1 or 2 days after a single injection of PBS (i.p.) or sACVR2B-Fc (10 mg/kg, i.p.) as previously described (Hulmi et al., 2013a; Figure 1A).

#### Two-Week sACVR2B-Fc Experiment in Healthy Mice

To study the short-term effects of sACVR2B-Fc administration in healthy skeletal muscle, mice were randomly divided into mice administered (i.p., 1–2 times per week) with (1) PBS or (2) sACVR2B-Fc as previously described (Hulmi et al., 2013a; Figure 1B).

#### Cancer Cachexia Experiment

Mice were randomized into one of the four weight-matched groups: (1) vehicle-treated (PBS) healthy control mice (CTRL), (2) C26 tumor-bearing mice administered with a vehicle (PBS) (C26 + PBS), (3) C26 tumor-bearing mice administered with sACVR2B-Fc before the C26 tumor formation and replaced by a vehicle (PBS) after the tumor formation (C26 + sACVR/b), and (4) C26 tumor-bearing mice continuously administered with sACVR2B-Fc throughout the experiment (C26 + sACVR/c). Cancer groups were inoculated subcutaneously (s.c.) with C26 colon carcinoma cells (5 × 10^5^ cells in ∼ 120 μl PBS) into their interscapular region. The vehicle (PBS) and sACVR2B-Fc (5 mg/kg in ∼ 100 μl PBS) were administered (i.p.) every fourth day (Figure 1C). The weight loss from day 10 to 11 after C26 inoculation strongly predicted survival (Nissinen et al., 2018) and thus the end-point was chosen to be 11 days after the C26 inoculation to represent the onset of cachexia. When the symptoms of cancer started to occur, mice were strictly and carefully monitored according to the end-point guidelines of animal experiments approved by the National Animal Experiment Board. However, none of the mice fulfilled the end-point criteria before the actual end-point.

#### sACVR2B-Fc Production

The production of the recombinant sACVR2B-Fc, which is similar but not totally identical with the original version generated by Lee et al. (2005), has been described in detail before (Hulmi et al., 2013a). Briefly, we fused a human IgG1 Fc domain with the ectodomain of human ACVR2B and the protein was expressed in Chinese hamster ovary cells grown in suspension culture.

### Tissue Collection and Processing

#### Tissue Collection

In the acute and 2-week experiments the mice were euthanized by cervical dislocation and gastrocnemius muscles were weighed and subsequently flash frozen in liquid nitrogen and stored at -80°C for further analysis. In the cancer experiment mice were euthanized by cervical dislocation after heart puncture under anesthesia [ketamine (Ketaminol^®^): ∼110–120 mg/kg + xylazine (Rompun^®^): 15–16 mg/kg]. Gastrocnemius, tibialis anterior (TA) and liver were weighed and flash frozen in liquid nitrogen and were stored at -80°C for further analysis.

#### RNA Extraction and cDNA Synthesis

In the cancer cachexia experiment, total RNA was extracted from gastrocnemius muscles with QIAzol and were purified with RNeasy Universal Plus kit (Qiagen). The quality of RNA was confirmed by spectrophotometry (NanoDrop; Thermo Fisher Scientific) and agarose gel electrophoresis. iScript^TM^ Advanced cDNA Synthesis Kit (Bio-Rad Laboratories) was used to reverse transcribe the RNA to cDNA. Both steps were conducted according to the manufacturer’s guidelines.

#### Protein Extraction and Content Measurement

Muscle and liver samples were homogenized in ice-cold buffer with protease and phosphatase inhibitors as previously described (Hulmi et al., 2013a; Nissinen et al., 2018). The total protein content was measured using the bicinchoninic acid protein assay (Pierce Biotechnology, Rockford, IL, United States) with an automated KoneLab analyzer (Thermo Fisher Scientific, Vantaa, Finland).

### Tissue Analyses

#### Real-Time-qPCR

mRNA expression levels were analyzed with RT-qPCR following standard procedures using iQ SYBR Supermix (Bio-Rad Laboratories) and CFX96 real-time PCR Detection system. The mRNA levels of *Chop*/*Ddit3* (assay ID qMmuCID0020314), *Lc3b* (assay ID qMmuCED0048150), and *P62* (assay ID qMmuCID0024517) were measured with pre-designed and pre-validated primers (Bio-Rad PrimePCR^TM^ SYBR Green assays). The protocol recommended by the manufacturer was used. The spliced variant mRNA level of X-box binding protein 1 (*Xbp1s*) was analyzed using SYBR green primers: forward: TGCTGAGTCCGCAGCAGGTG and reverse: CTGATGAGGTCCCCACTGACAGA (Invitrogen, United States). The protocol for the *Xbp1s* was initiated at 95°C which was followed by 39 cycles of denaturation at 95°C for 10 s, annealing at 61°C for 30 s and extension at 68°C for 30 s. mRNA expression levels were calculated from the exponential amplification phase using the efficiency corrected ΔΔCT method. *36b4* (Forward primer: 5′-GGCCCTGCACTCTCGCTTTC-3′, Reverse primer: 5′-TGCCAGGACGCGCTTGT-3′) was used as a house-keeping reference gene because it was unaffected by the cancer and the sACVR2B-Fc treatments as previously reported (*p*> 0.16) (Nissinen et al., 2018).

#### Western Blot

Western blot analyses were conducted in two laboratories and therefore slightly different protocols are provided below:

*LC3B, P62, Bcl-2, Beclin-1, p-ULK1^ser757^, ULK1, PERK, p-eIF2α^ser51^, total eIF2α, IRE1α, p-JNK54, JNK54, PDI, ATF4, Cleaved Caspase 3 and 12* as well as *HSP25, GRP78, and HSP47* were analyzed only from the cancer experiment by a protocol previously described in more detail (Hulmi et al., 2013a; Hentila et al., 2018). In short, muscle and liver homogenates mixed with Laemmli sample buffer + β-mercaptoethanol were heated at 95°C to denature proteins. Proteins were separated with SDS–PAGE and transferred to a PVDF membrane, blocked (5% fat-free milk in TBS-T) and incubated overnight at 4°C with primary antibodies. The membrane was then washed and incubated with secondary antibodies (Jackson ImmunoResearch Laboratories, West Grove, PA, United States) for 1 h followed by washing. Proteins were visualized by enhanced chemiluminescence (SuperSignal West Femto maximum sensitivity substrate, Pierce Biotechnology, Rockford, IL, United States) using a ChemiDoc XRS device and quantified with Quantity One software (version 4.6.3. Bio-Rad Laboratories, Hercules, CA, United States). The uniformity of the protein loading was confirmed by staining the membrane with Ponceau S and by re-probing the membrane with an antibody against GAPDH (Abcam, Cambridge, United Kingdom). The results were normalized to the mean of Ponceau S (strong band at ∼42 kDa) and GAPDH value.

*HSP60, HSP70, HSP90 TRX, and TxNIP* as well as *GRP78, HSP25, and HSP47* were analyzed from the acute experiments by a protocol as described previously (Atalay et al., 2004; Lappalainen et al., 2009). Briefly, protein extracts (20 μg protein per well) with molecular weight markers were electrophoresed on SDS/PAGE and transferred to a nitrocellulose membrane (Millipore, Bedford, MA, United States). The uniformity of the protein loading was confirmed by staining the membrane with Ponceau S and by re-probing the membrane with an antibody against Actin (Sigma, A-2066). Membranes were blocked with 5% fat-free milk solution at 37°C for 1 h and treated with monoclonal or polyclonal antibodies overnight at 4°C (StressGen, VIC, Canada; IMCO, Stockholm, Sweden; MBL International, Woburn, MA, United States; Sigma, St. Louis, MO, United States). Immunoblots were visualized by Odyssey (LI-COR Biosciences Inc., Lincoln, NB, United States) and quantified by Odyssey Software.

#### Antibodies

Antibodies for IRE1α (#3294), PDI (#3501), PERK (#3192), eIF2α (#5324) and its phosphorylated form at ser51 (#3398, p-eIF2α^ser51^) GRP78 (used in the cancer experiment, #3177), P62 (#5114), Bcl-2 (#3498), p-ULK1^ser757^ (#14202), ULK1 (#8054), p-JNK^Thr183/Tyr185^ (#4668), JNK (#9252), ATF4 (#11815), Beclin-1 (#3495), caspase 12 (#2202), and cleaved caspase 3 (#9661) were purchased from Cell Signaling Technology. GAPDH (ab9485) antibody was purchased from Abcam (Cambridge, United Kingdom). LC3I and LC3II were measured by antibody (L7543) that was purchased from Sigma-Aldrich (St. Louis, MO, United States). Monoclonal primary antibodies were used for the detection of heat shock protein 70 (HSP70, StressGen, SPA-810), heat shock protein 60 (HSP60, StressGen, SPA-806), heat shock protein 90 (HSP90, StressGen, SPA-835), heat shock protein 47 (HSP47, StressGen, SPA-470) and thioredoxin interacting protein (TXNIP and MBL). Polyclonal primary antibodies were used to detect thioredoxin (TRX, IMCO, and ATRX-06), actin (Sigma, A-2066), heat shock protein 25 (HSP25, StressGen, and SPA-801), glucose-regulated protein 78 (GRP78, StressGen, SPA-826, used in the acute experiments). Horseradish peroxidase conjugated IgG secondary antibodies were used (Jackson ImmunoResearch Laboratories, PA, United States and StressGen and Zymed, San Francisco, CA, United States).

#### Analysis of Protein Carbonyls

In the acute and 2 weeks experiments protein carbonyls were analyzed by western blot technique after derivatization with 2,4-dinitrophenyl hydrazine immediately before the electrophoresis, as previously described (Hulmi et al., 2016). In the C26 cancer experiment, the principle of the measurement was the same as with acute and 2 weeks experiment. However, the measurement was carried out with a commercial OxyBlot Protein Oxidation Detection kit (Merck Millipore, S1750) according to manufacturer’s instructions as previously described (Hentila et al., 2018).

#### Glutathione Assays

After gastrocnemius muscle homogenization, total glutathione (TGSH) was measured spectrophotometrically by an oxidized glutathione (GSSG) reductase recycling method as described earlier (Lappalainen et al., 2009; Hulmi et al., 2016). The rate of change in absorbance at 412 nm was monitored with a double-beam spectrophotometer at room temperature and tissue concentrations were estimated by linear regressions from the standard curve.

### Statistical Methods

The main effect was analyzed by one-way analysis of variance (ANOVA) or the Kruskal-Wallis test, followed by Holm-Bonferroni corrected LSD or Mann-Whitney U *post hoc* tests depending on the distribution of the data (Shapiro-Wilk). The effect of 2-week sACVR2B-Fc (sACV 2wk vs. PBS 2wk) administration was examined with Student’s *t* test or Mann Whitney U if data were not normally distributed. The C26 cancer effect (CTRL vs. C26 + PBS and CTRL vs. C26 groups pooled) was analyzed with Student’s *t* test or the Mann-Whitney *U*-test when data were not normally distributed. Correlations were analyzed using Pearson’s product-moment coefficient.

PASW statistics version 24.0 was used for statistical analyses (SPSS, Inc., Chicago, IL, United States). The level of significance was set at *P* ≤ 0.05. Data are expressed as means ± SE.

## Results

### Background Results

This is a follow-up study on our two previous studies. In brief, in Nissinen et al. (2018) we showed that C26 tumor implantation resulted in muscle and fat wasting and increased hepatic protein synthesis as well as acute phase response, a cytokine-induced early defense mechanism (Cray et al., 2009). Treating mice with sACVR2B-Fc increased muscle mass and protein synthesis in healthy mice (Hulmi et al., 2013a) and prevented muscle loss and prolonged survival in tumor-bearing mice without affecting the tumor size when sACVR2B-Fc was administered continuously before and after the C26 cell inoculation (Nissinen et al., 2018).

### Two-Week sACVR2B-Fc Administration Induces Unfolded Protein Response

**FIGURE 2:**
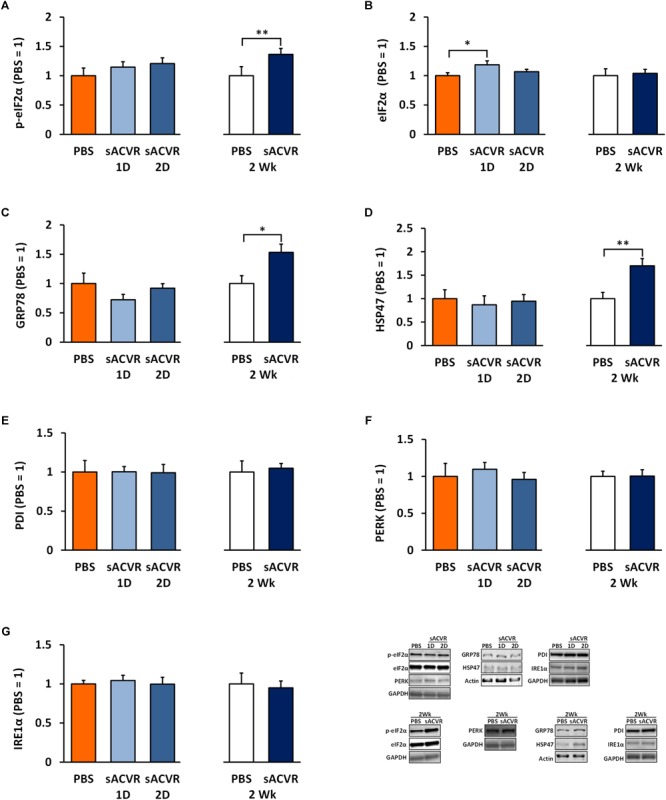
Unfolded protein response (UPR) markers in healthy skeletal muscle in mice that were administered with soluble activin receptor type 2B (sACVR) or vehicle (PBS). Muscles were collected 1 or 2 days after a single sACVR administration or after 2 weeks of administration. **(A)** p-eIF2α^ser51^, **(B)** total eIF2α protein, **(C)** GRP78 protein, **(D)** HSP47 protein, **(E)** PDI protein, **(F)** PERK protein, **(G)** IRE1α protein; *n* = 6–7 in all groups except *n* = 9–11 in sACVR 2 Wk group. The symbol ^∗^ depicts statistical significance *p* < 0.05 whereas the symbol ^∗∗^ depicts statistical significance *p* < 0.01. Data is expressed as means ± SE. Representative blots were cropped from the original blot images (Supplementary Figures 6–8). Representative blots with same IDs and analyzed in the same run are aligned on top of each other and share the same representative control blot (GAPDH or actin).

The protein content of UPR indicators and ER-resident chaperones were unchanged 1 and 2 days after the single sACVR2B-Fc administration (Figures 2A–G) when muscle protein synthesis was greatly induced (Hulmi et al., 2013b). Later, after 2 weeks of sACVR2B-Fc administration, the phosphorylation of eIF2α^Ser51^ (*p* < 0.05) was increased without changes in total eIF2α (Figures 2A,B). In addition, ER resident chaperones GRP78 (*p* < 0.05) and HSP47 (*p* < 0.01) were increased (Figures 2C,D), suggesting partial induction of UPR by 2-week sACVR2B-Fc administration, while other UPR indicators were unchanged (PERK, PDI, and IRE1α) (Figures 2E–G).

### Protein Carbonylation and the Reduced Form of Glutathione Are Increased by sACVR2B-Fc Administration

**FIGURE 3:**
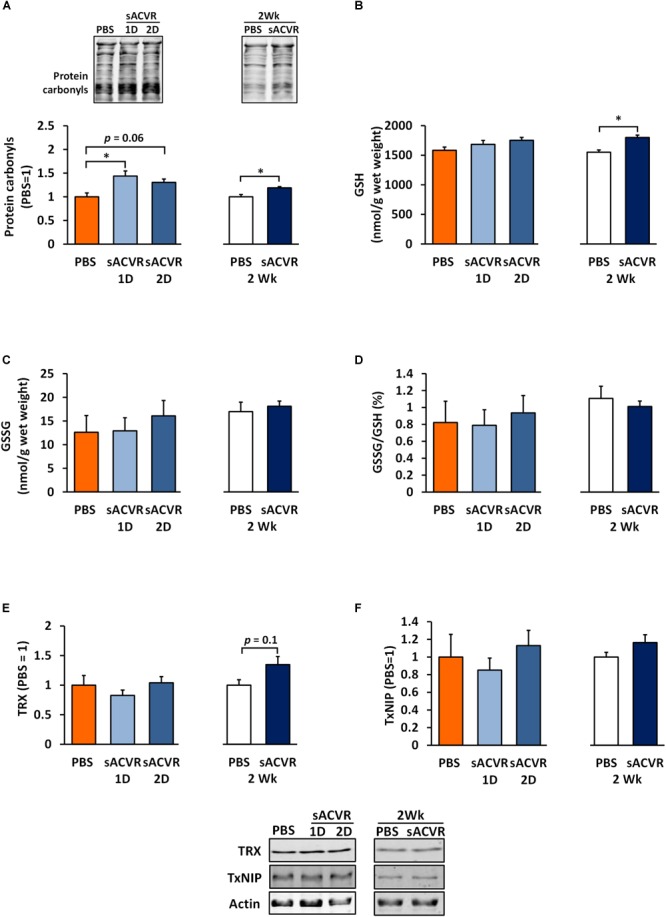
Redox-balance markers in healthy skeletal muscle in mice that were administered with soluble activin receptor type 2B (sACVR) or vehicle (PBS). Muscles were collected 1 or 2 days after a single sACVR administration or after 2 weeks of administration. **(A)** Protein carbonyls, **(B)** reduced glutathione (GSH), **(C)** oxidized glutathione (GSSG), **(D)** ratio of oxidized glutathione to reduced glutathione (GSSG/GSH), **(E)** TRX protein, **(F)** TxNIP protein; *n* = 5–6 in all groups except *n* = 8–11 in sACVR 2 Wk group. The symbol ^∗^ depicts statistical significance *p* < 0.05. Data is expressed as means ± SE. Representative blots were cropped from the original blot images (Supplementary Figure 7). Representative blots of TRX and TxNIP share the same IDs and were analyzed in the same run and are thus aligned on top of each other and share the same representative control blot (actin). Protein carbonyls were normalized to total protein loading.

**FIGURE 4:**
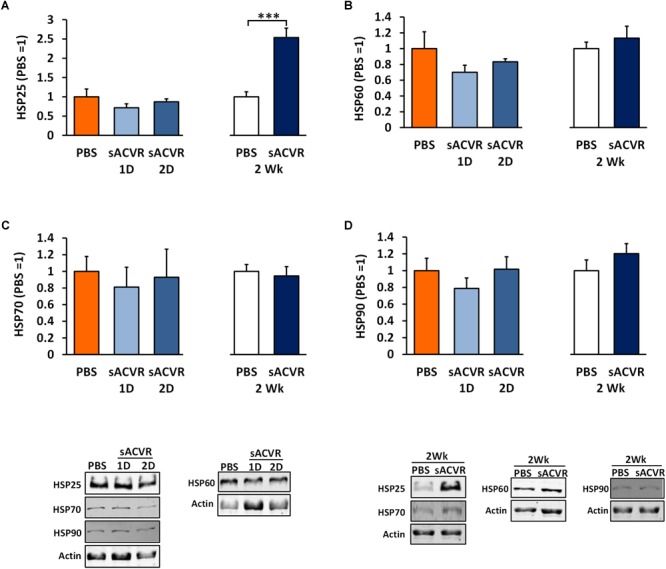
Heat shock protein (HSP) response markers in healthy skeletal muscle in mice that were administered with soluble activin receptor type 2B (sACVR) or vehicle (PBS). Muscles were collected 1 or 2 days after a single sACVR administration or after 2 weeks of administration. **(A)** HSP25, **(B)** HSP60, **(C)** HSP70, and **(D)** HSP90; *n* = 6–7 in all groups except *n* = 10–11 in sACVR 2 Wk group. The symbol ^∗∗∗^ depicts statistical significance *p* < 0.001. Data is expressed as means ± SE. Representative blots were cropped from the original blot images (Supplementary Figure 8). Representative blots with same IDs and analyzed in the same run are aligned on top of each other and share the same representative control blot (actin).

As a marker of oxidative damage, protein carbonyls were increased 1 and 2 days after the sACVR2B-Fc administration in skeletal muscle (Day 1: *p* < 0.05, Day 2: *p* = 0.06, Figure 3A) and remained increased after 2 weeks of sACVR2B-Fc administration (*p* < 0.05) (Figure 3A). Possibly as a delayed response to increased oxidative stress, there was an increase in reduced glutathione (*p* < 0.05, Figure 3B) and a trend for increased TRX protein content (*p* = 0.10, Figure 3E) after 2 weeks of sACVR2B-Fc administration without changes in the protein content of TxNIP (Figure 3F). Oxidized glutathione (GSSG) concentration (Figure 3C) and the ratio of oxidized and reduced glutathione (GSSG/GSH) (Figure 3D) were unchanged by the sACVR2B-Fc administration in all the time-points. Of the heat shock response indicators, 2-week sACVR2B-Fc administration increased only the protein content of small heat shock protein 25 (HSP25) at 2 weeks (*p* < 0.001, Figure 4A) without changes in larger HSPs 60, 70, and 90 at any time-point (Figures 4B–D).

### Decreased Glutathione Levels in C26 Tumor-Bearing Mice Are Restored by Continued sACVR2B-Fc Administration

**FIGURE 5:**
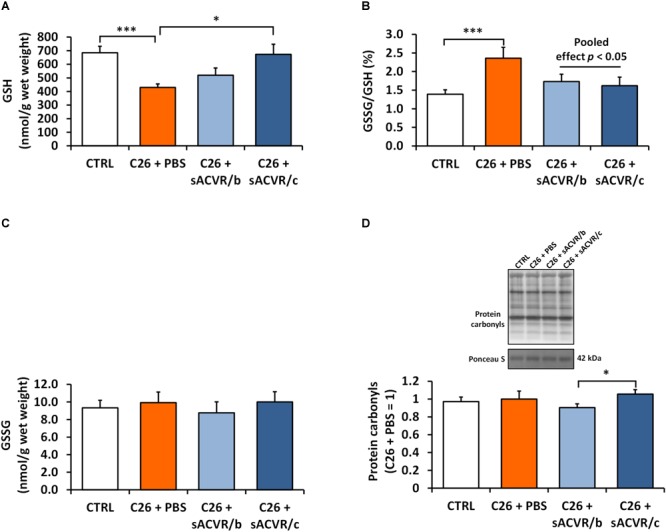
Redox balance markers in skeletal muscle of C26 tumor-bearing mice. **(A)** Reduced glutathione (GSH), **(B)** ratio of oxidized to reduced glutathione (GSSG/GSH), **(C)** oxidized glutathione (GSSG), and **(D)** protein carbonyls. CTRL, vehicle-treated (PBS) healthy control mice, *n* = 7–9. C26 + PBS = C26 tumor-bearing mice administered with a vehicle (PBS), *n* = 7. C26 + sACVR/b = C26 tumor-bearing mice administered with sACVR before the tumor formation and replaced by a vehicle (PBS) after the tumor formation, *n* = 7. C26 + sACVR/c = C26 tumor-bearing mice continuously administered with sACVR throughout the experiment, *n* = 8. The symbol ^∗^ depicts statistical significance *p* < 0.05 whereas the symbol ^∗∗∗^ depicts statistical significance *p* < 0.001. For the pooled effect sACVR administered groups were pooled. Data is expressed as means ± SE. Protein carbonyls were normalized to Ponceau S staining (strongest band at ∼42 kDa). Representative protein carbonyl blot was cropped from the original blot image (Supplementary Figure 10).

Next, we investigated the effects of the C26 tumor that induced cachexia and sACVR2B-Fc administration that alleviated cachexia. As a marker of skeletal muscle redox balance, the reduced form of glutathione was decreased in PBS-treated tumor-bearing mice compared with healthy controls (*p* < 0.001, Figure 5A). This was accompanied by increased ratio of oxidized glutathione and reduced glutathione (GSSG/GSH), suggesting increased oxidative stress (*p*< 0.001, Figure 5B). Interestingly, sACVR/c administration prevented the decrease in reduced glutathione levels (*p* < 0.05), thus decreasing the GSSG/GSH ratio (pooled sACVR effect, *p* < 0.05) (Figure 5B). Oxidized glutathione concentration (GSSG) (Figure 5C) and a marker of oxidative damage/stress, protein carbonyl content were unaltered by cancer and sACVR2B-Fc treatment (Figure 5D).

### Cancer Cachexia Is Associated With Decreased HSP47, p-eIF2α, and p-JNK54 of the UPR Indicators in Muscle

**FIGURE 6:**
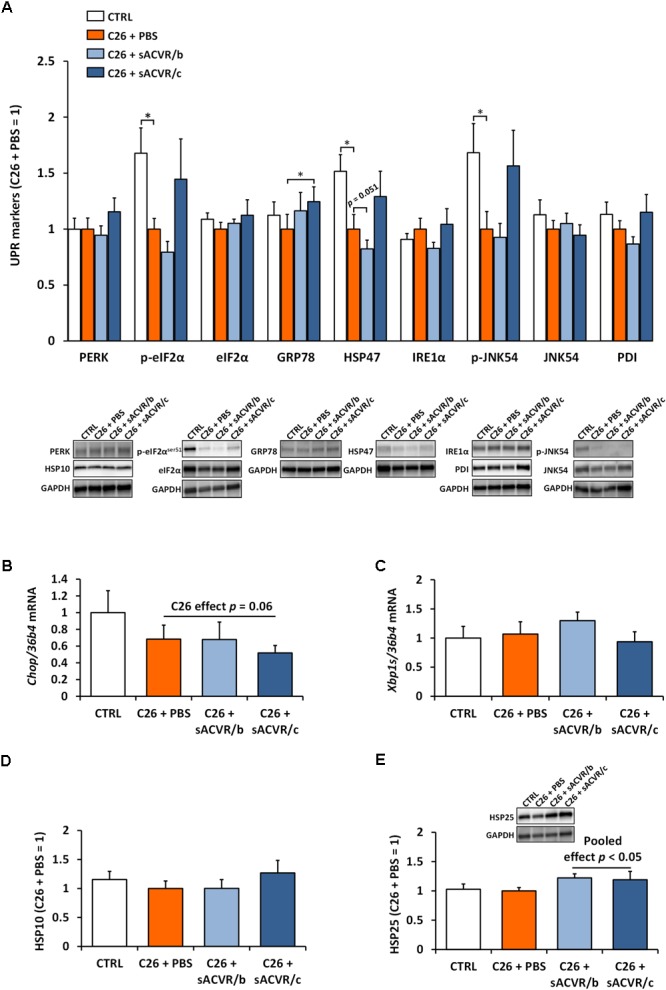
Unfolded protein response (UPR) and heat shock protein (HSP) response markers in skeletal muscle of C26 tumor-bearing mice. **(A)** Protein content of UPR markers (PERK, p-eIF2α^ser51^, eIF2α, GRP78, HSP47, IRE1α, p-JNK54, JNK54, and PDI), **(B)** mRNA level of *Chop*, and **(C)** of spliced *Xbp1 (Xbp1s)*. **(D)** Protein content of HSP10 and **(E)** HSP25. CTRL, vehicle-treated (PBS) healthy control mice, *n* = 7. C26 + PBS = C26 tumor-bearing mice administered with a vehicle (PBS), *n* = 7. C26 + sACVR/b = C26 tumor-bearing mice administered with sACVR before the tumor formation and replaced by a vehicle (PBS) after the tumor formation, *n* = 7. C26 + sACVR/c = C26 tumor-bearing mice continuously administered with sACVR throughout the experiment, *n* = 8. The symbol ^∗^ depicts statistical significance *p* < 0.05. For the C26 effect all the tumor-bearing groups were pooled. For the sACVR pooled effect both of the sACVR groups were pooled. Data is expressed as means ± SE. Representative blots were cropped from the original blot images (Supplementary Figures 9, 10). Representative blots with same IDs and analyzed in the same run are aligned on top of each other and share the same representative control blot (GAPDH).

Of the UPR markers, C26 cancer decreased the phosphorylation of eIF2α at Ser^51^ and the phosphorylation of JNK54 at Thr^183^/Tyr^185^, as well as decreased the levels of HSP47 protein in skeletal muscle (*p*< 0.05, Figure 6A). In addition, the mRNA level of the pro-apoptotic indicator *Chop* tended to be decreased when tumor-bearing groups were pooled (*p* = 0.06, Figure 6B). Other UPR indicators remained unaltered by C26 cancer (Figures 6A,C). The continued sACVR2B treatment (sACVR/c) increased GRP78 protein (*p* < 0.05) compared to PBS-administered mice and tended to increase p-eIF2α^Ser51^ (*p* = 0.18), HSP47 (*p* = 0.11), and p-JNK54 (*p* = 0.14) to healthy control levels (Figure 6A). A mitochondrial UPR marker HSP10 and HSP25 were unaltered by C26 cancer (Figures 6D,E). However, when sACVR2B treated groups were pooled, HSP25 protein was increased compared to PBS-treated C26 tumor-bearing mice (*p* < 0.05, Figure 6E).

### Selective Hepatic UPR Is Induced in Tumor-Bearing Mice

**FIGURE 7:**
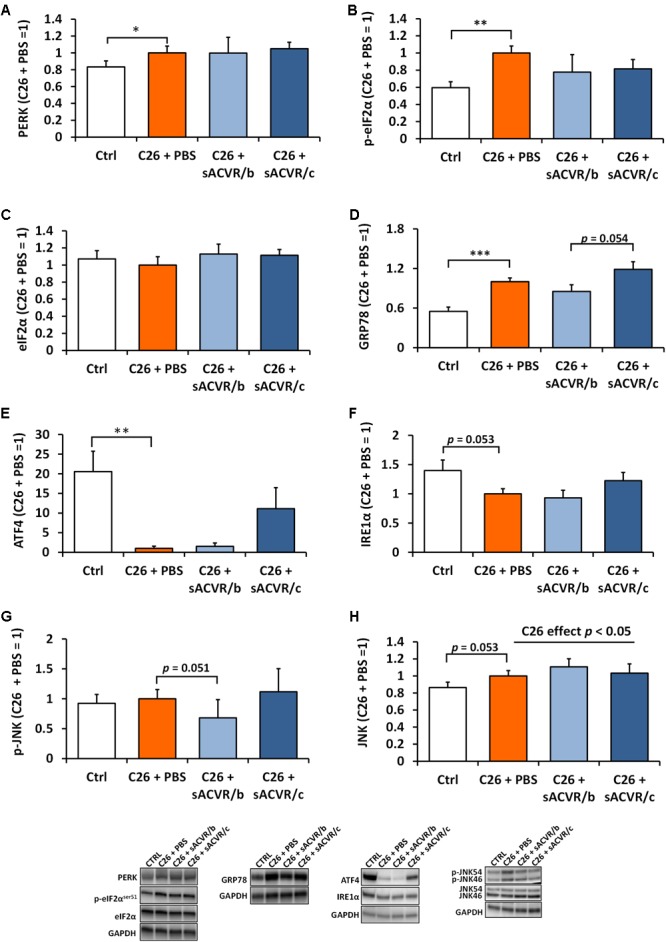
Unfolded protein response (UPR) markers in the liver of C26 tumor-bearing mice. **(A)** PERK protein, **(B)** p-eIF2α^ser51^, **(C)** eIF2α, **(D)** GRP78 protein, **(E)** ATF4 protein, **(F)** IRE1α protein, **(G)** mean of p-JNK46 and p-JNK54, and **(H)** mean of total JNK46 and JNK54 protein. CTRL, vehicle-treated (PBS) healthy control mice, *n* = 9. C26 + PBS = C26 tumor-bearing mice administered with a vehicle (PBS), *n* = 7. C26 + sACVR/b = C26 tumor-bearing mice administered with sACVR before the tumor formation and replaced by a vehicle (PBS) after the tumor formation, *n* = 7. C26 + sACVR/c = C26 tumor-bearing mice continuously administered with sACVR throughout the experiment, *n* = 8. The symbol ^∗^ depicts statistical significance *p* < 0.05, the symbol ^∗∗^ depicts statistical significance *p* < 0.01 and the symbol ^∗∗∗^ depicts statistical significance *p* < 0.001. For the C26 effect, tumor-bearing groups were pooled. Data is expressed as means ± SE. Representative blots were cropped from the original blot images (Supplementary Figures 11, 12). Representative blots with same IDs and analyzed in the same run are aligned on top of each other and share the same representative control blot (GAPDH).

In the liver, the UPR indicators PERK (*p* < 0.05), p-eIF2α^Ser51^ (*p* < 0.01) (no change in total eIF2α) and GRP78 (*p* < 0.001) were increased by C26 cancer (Figures 7A–D). However, ATF4 protein content was greatly decreased (*p*< 0.01, Figure 7E) and there was a trend for decrease in IRE1α (*p* = 0.053, Figure 7F). Phosphorylation of JNK was unaltered by cancer (Figure 7G) but total JNK was increased in tumor-bearing mice when groups were pooled (*p* < 0.05, Figure 7H). sACVR2B-Fc administration had no significant effect on any of the variables. Pro caspase 12 was decreased (*p* < 0.05) by C26 cancer but its cleaved form was unchanged (Supplementary Figure 1). In addition, cleaved caspase 3 (Supplementary Figure 1) was unchanged, suggesting that apoptosis was not activated at this time point.

### Autophagy-Lysosome Pathway Is Induced in Skeletal Muscle and Liver of Tumor-Bearing Mice

**FIGURE 8:**
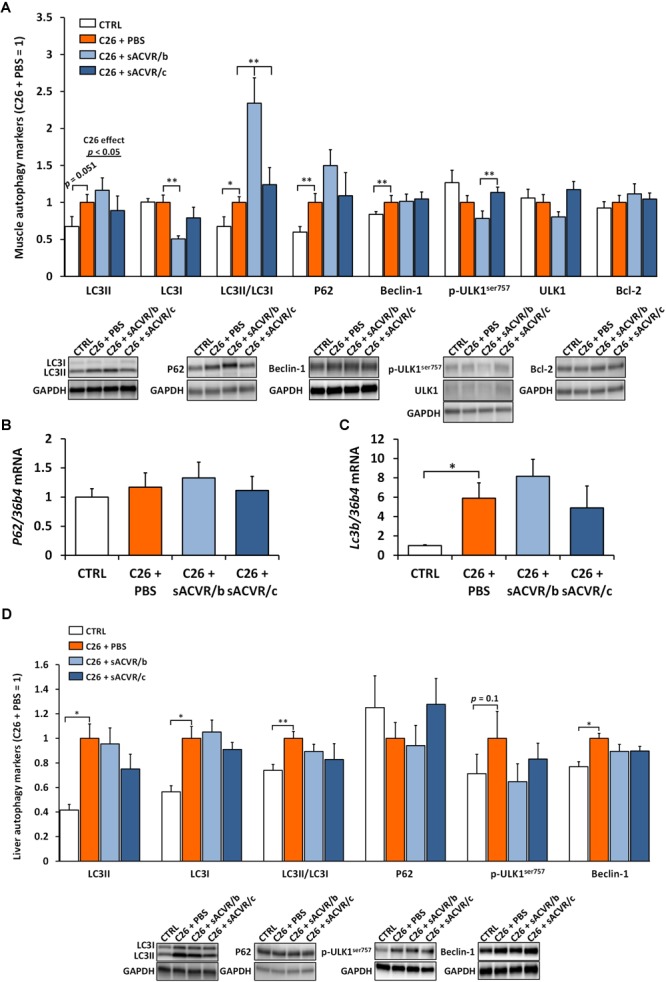
Autophagy markers in skeletal muscle and the liver of C26 tumor-bearing mice. **(A)** Protein content of autophagy markers in skeletal muscle (LC3II, LC3I, LC3II/LC3I, P62, Beclin-1, p-ULK1^ser757^, total ULK1, and Bcl-2). The mRNA level of **(B)** *P62* and **(C)** *Lc3b* in skeletal muscle. **(D)** Protein content of autophagy markers in the liver (LC3II, LC3I, LC3II/LC3I, P62, p-ULK1^ser757^, and Beclin-1). CTRL, vehicle-treated (PBS) healthy control mice, *n* = 7–9. C26 + PBS = C26 tumor-bearing mice administered with a vehicle (PBS), *n* = 7. C26 + sACVR/b = C26 tumor-bearing mice administered with sACVR before the tumor formation and replaced by a vehicle (PBS) after the tumor formation, *n* = 7. C26 + sACVR/c = C26 tumor-bearing mice continuously administered with sACVR throughout the experiment, *n* = 8. The symbol ^∗^ depicts statistical significance *p* < 0.05 and the symbol ^∗∗^ depicts statistical significance *p* < 0.01. For the C26 effect, tumor-bearing groups were pooled. Data is expressed as means ± SE. Representative blots were cropped from the original blot images (Supplementary Figures 9, 11, 12). Representative blots with same IDs and analyzed in the same run are aligned on top of each other and share the same representative control blot (GAPDH).

In skeletal muscle, lipidated LC3 (LC3II) (*p* = 0.051) and the ratio of LC3II to LC3I (*p* < 0.05) which can be used as a marker of autophagosome content, were both increased by the C26 cancer (Figure 8A). In addition, Beclin-1, involved in autophagy induction was increased by C26 cancer (*p* < 0.01, Figure 8A). Moreover, the protein content of P62, which acts as an adaptor protein sequestering cellular compartments that are to be degraded by autophagy–lysosome pathway, was also increased by C26 cancer (*p* < 0.01, Figure 8A). *Lc3b* mRNA (*p* < 0.05, Figure 8C) was increased by cancer whereas the protein content of LC3I, p-ULK1^Ser757^ as well as total ULK1 and Bcl-2 and *p62* mRNA were unaltered (Figures 8A,B).

LC3II (*p* < 0.05), LC3II/I (*p* < 0.01), and Beclin-1 (*p* < 0.05) increased in the liver by the C26 cancer without changes in p-ULK1^Ser757^ and P62 protein (Figure 8D). Contrary to skeletal muscle, LC3I (*p* < 0.05) increased in the liver by C26 cancer. The discontinued administration of sACVR2B-Fc further increased LC3II/I in skeletal muscle compared to other tumor-bearing groups (*p* < 0.01, Figure 8A). Otherwise there were no systematic effects of sACVR2B-Fc administration on the autophagy indicators in skeletal muscle or in the liver (Figures 8A–D).

## Discussion

We demonstrated that muscle hypertrophy induced by blocking ACVR2B ligands increased markers of unfolded protein response (UPR) and the reduced form of glutathione. On the other hand, experimental C26 cancer cachexia was accompanied by decreased levels of reduced glutathione, indicating a decline in antioxidant defense capacity in skeletal muscle. Interestingly, blocking ACVR2B ligands restored this decline. Moreover, we showed that selective UPR was induced in the liver, but not in skeletal muscle during the onset of rapid weight loss in experimental cancer.

We (Nissinen et al., 2018) and others (Cai et al., 2004; Zhou et al., 2010) have previously shown that the prevention of cancer cachexia without any effect on tumor growth improves survival in experimental mouse models. This result suggests a possible causality between the prevention of muscle loss and improved survival in cachexia, but its underlying mechanisms are unknown. More specifically, we showed that a soluble growth factor receptor, namely sACVR2B-Fc, improved survival in C26 cancer cachexia only when the treatment continued after the tumor formation, whereas a discontinued prophylactic treatment had no positive survival effects (Nissinen et al., 2018). In the present study, we further investigated the tumor-bearing mice and elucidated the role of biological processes regulating protein homeostasis. We observed altered redox balance, which shifted toward increased oxidative stress manifested by decreased levels of reduced glutathione (GSH) resulting in an increased ratio of oxidized to reduced glutathione (GSSG/GSH) in the skeletal muscle of tumor-bearing mice. The result is consistent with previous studies in which hallmarks of altered redox state were manifested by decreased levels of GSH (Ham et al., 2014) and a depletion of antioxidant peptides (glutathione, anserine, and carnosine), accompanied by a shift of the ratio of mannitol and mannose toward the oxidized form (Der-Torossian et al., 2013) in the skeletal muscle of tumor-bearing mice. Interestingly, together with improving survival (Nissinen et al., 2018), only the continued blocking of ACVR2B ligands restored glutathione levels in cachectic skeletal muscle in line with the levels in the healthy mice. Moreover, blocking ACVR2B ligands increased the reduced form of glutathione in healthy mice as well, suggesting that the effect is not limited to cachectic muscle only. This supports the earlier study in which the absence of normal myostatin signaling *per se* increased GSH concentration (Ploquin et al., 2012). We also observed that muscle GSH concentration correlated positively with adipose tissue mass, skeletal muscle mass and change in body mass (last 2 days of the experiment) as well as negatively with tumor mass, suggesting that skeletal muscle GSH depletion is associated with the severity of the cachexia (Supplementary Figures 2A–D). Indeed, in a previous study, administration of glycine, which is a precursor for glutathione, improved muscle function and inhibited muscle wasting in C26 cachexia (Ham et al., 2014), suggesting that the reduced GSH may have an impact on the cachexia progression and restoring the redox balance may be beneficial.

The increase in GSH may also be a compensatory mechanism for increased oxidative damage/stress after a single or repeated sACVR administration. Unlike in healthy mice, protein carbonyls were, however, unchanged in tumor-bearing mice with or without blocking ACVR2B ligands. The accumulation of oxidatively damaged proteins is also known to induce proteasome activity intended to achieve their removal (Radak et al., 2000). Therefore, no difference in oxidatively modified protein levels may be explained by their increased removal. To support this assumption we observed increased markers of protein degradation (Nissinen et al., 2018) and in the current study increased markers of autophagy. Thus, a possible increase of oxidative protein modification cannot be discarded in tumor-bearing mice. Heat shock proteins are known to be induced, for example, by oxidative and metabolic stress (Niforou et al., 2014). We did not observe induction of the expression HSPs 60, 70, and 90 in healthy skeletal muscle, which does not support the assumption that sACVR2B-Fc administration increases cellular and especially oxidative stress. However, the expression of HSP25, which is also known to protect cells from oxidative stress (Escobedo et al., 2004) was also upregulated in healthy and cachectic sACVR administered mice, the effect being more profound in healthy muscle. This result is in line with previous literature in which skeletal muscle hypertrophy was observed (Huey, 2006; Frier and Locke, 2007; Huey et al., 2010), suggesting that induction of HSP25 regulates muscle homeostasis during rapid muscle hypertrophy. We analyzed only HSP content to assess heat shock response and not their subcellular localization, which is a key feature of their function (Paulsen et al., 2007, 2009). Future studies are warranted to investigate the effect of blocking ACVR2B ligands on oxidative stress and redox regulation as well as HSP localization in more detail.

To further investigate muscle protein homeostasis, we analyzed several indicators of unfolded protein response (UPR), a process that is also known to be induced by ER stress and oxidative stress (Cao and Kaufman, 2014). We observed that ER stress and UPR are not induced at the onset of C26 induced cachexia and, if anything, UPR may instead be downregulated. These observations are in line with decreased p-eIF2α in C26 tumor-bearing mice (Penna et al., 2010). However, they are contrary to a previous study in which UPR in skeletal muscle was upregulated in two other commonly used experimental animal models of cancer cachexia: LLC and Apc^min/+^ mice. Interestingly, UPR seems to protect muscles from further wasting in these experimental cancer models (Bohnert et al., 2016; Gallot et al., 2018). The decreased UPR indicators were associated with the severity of cachexia because the downregulated UPR indicators (p-eIF2α, HSP47 and p-JNK) correlated positively with the change in body mass between the last 2 days of the experiment and negatively with the tumor mass (Supplementary Figures 3A–F). Alternatively, the decreased levels of UPR indicators in skeletal muscle that we observed in tumor-bearing mice could possibly be explained by decreased overall protein synthesis (Nissinen et al., 2018), which would reduce the demand of the folding of the nascent newly synthesized proteins. To support this, p-eIF2α, HSP47, and p-JNK correlated positively with muscle protein synthesis (Supplementary Figures 4A–C). Another possible mechanism downregulating these indicators may be a decrease in physical activity that we previously reported (Nissinen et al., 2018) because HSP47 has been shown to decline during muscle unloading (Oguro et al., 2003) and phosphorylation of JNK has been shown to be inducible by resistance exercise (Hentila et al., 2018) and mechanical strain (Martineau and Gardiner, 2001).

Interestingly, many of the downregulated UPR markers in cancer were or tended to be increased in mice that received continued sACVR2B-Fc treatment, compared with other tumor-bearing mice. In healthy mice, 2-week sACVR2B-Fc administration increased the same UPR markers (GRP78, HSP47, and p-eIF2α) that also tended to be upregulated in sACVR2B-Fc administered C26 tumor-bearing mice. This suggests that these UPR indicators are upregulated more ubiquitously by sACVR2B-Fc administration. However, UPR indicators were not induced acutely 1 or 2 days after a single administration of sACVR2B-Fc, even though the protein synthesis was greatly induced, as we previously reported (Hulmi et al., 2013a). This is contrary to another stimulus, increasing protein synthesis, resistance exercise (RE), which has been shown to induce UPR 1 and 2 days after the RE bout (Ogborn et al., 2014; Hentila et al., 2018). Thus, it is suggested that UPR activation following sACVR2B-Fc ligand blocking is delayed compared with a RE bout. Previously, activation of UPR has been shown to inhibit mTORC1 signaling (Deldicque et al., 2010, 2011) and was suggested to act as a molecular break, suppressing protein synthesis during rapid muscle growth (Hamilton et al., 2014). Indeed, based on growth curves, the rate of muscle growth seemed to reach a plateau after 10 days during the sACVR2B-Fc administration (Hulmi et al., 2013a), which might be attributed to the induction of UPR and consequently blunted muscle growth.

We previously reported increased hepatic protein synthesis and induction of acute phase response (APR), suggesting altered protein homeostasis in the liver of tumor-bearing mice (Nissinen et al., 2018). Thus, we also analyzed UPR indicators in the liver of the tumor-bearing mice. In contrast to skeletal muscle, PERK, p-eIF2α, and GRP78 were increased by C26, whereas ATF4 protein content was strongly decreased. These results suggest that specific branches of UPR are activated in the liver of the tumor-bearing mice while some indicators may respond with a strong decrease. In a previous study, specific UPR markers have been associated with the severity of the cachexia: the pro-apoptotic indicators were especially associated with the progression of cachexia (Narsale et al., 2015). To analyze the maladaptive UPR branch that drives apoptosis, we analyzed the content of the ER stress-specific apoptosis marker caspase 12 (Szegezdi et al., 2006) from the livers of tumor-bearing mice. Interestingly, the content of the cleaved, active caspase 12 was unchanged, but the uncleaved pro caspase 12 was decreased in the liver of tumor-bearing mice. In addition, another ER-stress-associated pro-apoptotic indicator, JNK phosphorylation (Urano et al., 2000; Szegezdi et al., 2006) and the apoptosis marker cleaved caspase 3 were unchanged. This suggests that ER-stress-driven apoptosis was not induced in the liver of tumor-bearing mice. Nevertheless, the hepatic UPR markers GRP78 and ATF4 correlated positively with the change in body mass (the last 2 days of the experiment) (Supplementary Figures 5A,B) and ATF4 correlated negatively with the tumor mass (Supplementary Figure 5C), suggesting that also some of the hepatic UPR indicators may be associated with the severity of cachexia.

As a third biological process that is connected to redox balance (Lee et al., 2012) and UPR (Yorimitsu et al., 2006) and which contributes to the muscle protein homeostasis, we investigated the indicators of the autophagy-lysosome pathway that is responsible for the degradation and recycling of unnecessary and/or damaged proteins and organelles (Sandri, 2013) from the tumor-bearing mice. We observed increased content of lipidated LC3, increased ratio of LC3II and LC3I (LC3II/LC3I), *Lc3b* mRNA and Beclin-1 in the skeletal muscle of C26 tumor-bearing mice, which indicates increased autophagosome content and the induction of autophagy, respectively. These results were accompanied by increased P62 protein and unchanged *P62* mRNA levels, suggesting decreased clearance of autophagosomes (Klionsky et al., 2016). Our results are consistent with the previous studies conducted with experimental animal models and also in cachectic humans (Penna et al., 2013; Aversa et al., 2016; Molinari et al., 2017), clearly indicating that autophagy is induced in cachectic muscle and that the clearance of the autophagosomes may be inhibited. Even though the continued sACVR2B-Fc treatment previously inhibited the muscle wasting in the tumor-bearing mice (Nissinen et al., 2018) and has decreased markers of autophagy in healthy mice (Hulmi et al., 2013b), the novelty of our present study was that the sACVR2B-Fc treatment did not inhibit the increase in autophagy that was observed 11 days after the C26 inoculation. These results suggest that autophagy contributes to the muscle wasting together with the ubiquitin-proteasome system, which was also upregulated in the muscle of the C26 tumor-bearing mice despite the sACVR treatment (Nissinen et al., 2018). In the liver, as in the skeletal muscle, lipidated LC3 content and LC3II/LC3I were increased in C26 tumor-bearing mice but not P62, suggesting increased autophagosome content without impairment in the autophagic flux. Our results suggest that cancer cachexia induces aberration in the hepatic proteostasis, which is compensated by the induction of autophagy and UPR without any evident ER-stress related apoptosis. The induction of autophagy may also be explained by decreased feed intake, a known stimulus for autophagy activation (Sandri, 2010, 2013).

In conclusion, experimental cancer cachexia decreased the anti-oxidant defense capacity manifested by decreased glutathione content in skeletal muscle. However, sACVR2B-Fc administration increased glutathione in both healthy and cachectic muscle, suggesting that it increases anti-oxidant defense capacity in skeletal muscle. Based on the correlations of the data, we suggest that alterations in the processes contributing to protein homeostasis are associated with the severity of cachexia. The induction of UPR in the liver but not in skeletal muscle indicates that the protein homeostasis is altered in a tissue-specific manner in C26 cancer cachexia.

## Author Contributions

JJH, JH, and TN designed the cancer experiments. JJH and OR designed the acute study. TN and JH carried out the cancer experiments. JJH carried out the acute sACVR2B-Fc experiments with the healthy mice. MS and SL assisted in the *in vivo* experiments. JH drafted the manuscript with the help from JJH, MA, and TN. JH carried out the analysis with the help from TN, MA, and AK. AP and OR designed and produced the recombinant sACVR2B-Fc used in the study. All authors read and approved the final manuscript.

## Conflict of Interest Statement

The authors declare that the research was conducted in the absence of any commercial or financial relationships that could be construed as a potential conflict of interest.
