# Supplementary material for: Activin Receptor Ligand Blocking and Cancer Have Distinct Effects on Protein and Redox Homeostasis in Skeletal Muscle and Liver
Source: Front Physiol. 2019 Jan 18;9:1917. doi: 10.3389/fphys.2018.01917 (PMC6345696; doi:10.3389/fphys.2018.01917)
Supplement: Supplementary file 1 [file Image_1.pdf]

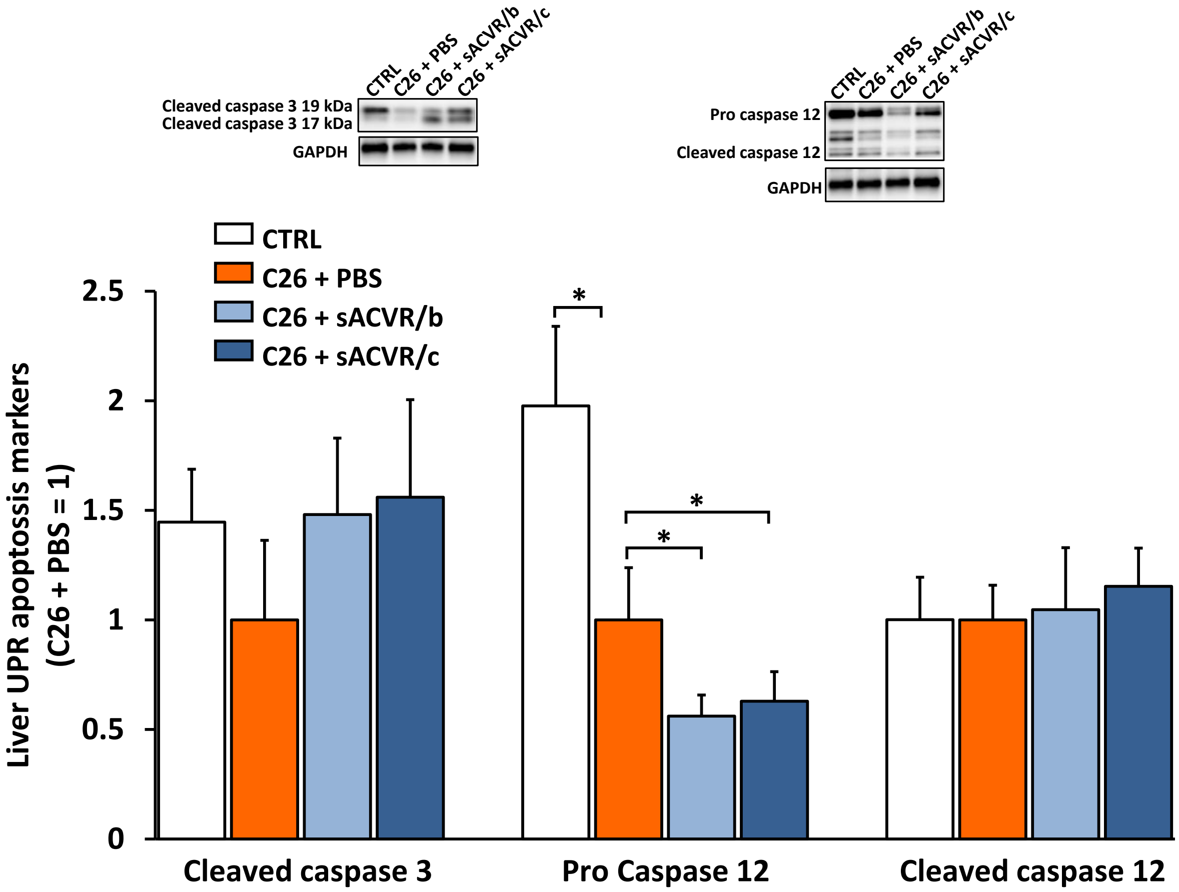

Supplementary figure 1. Unfolded protein response (UPR) related apoptosis markers in the liver (mean of 2 isoforms of cleaved caspases (17 and 19 kDa), pro caspase 12 (55 kDa) and cleaved caspase 12 (42 kDa)). CTRL = vehicle-treated (PBS) healthy control mice, *n* = 9. C26 + PBS = C26 tumor-bearing mice administered with a vehicle (PBS), *n* = 7. C26 + sACVR/b = C26 tumor-bearing mice administered with sACVR before the tumor formation and replaced by a vehicle (PBS) after the tumor formation, *n* = 7. C26 + sACVR/c = C26 tumor-bearing mice continuously administered with sACVR throughout the experiment, *n* = 8. The symbol * depicts statistical significance $p < 0.05$. Data is expressed as means ± SE. Representative blots were cropped from the original blot images (**Supplementary figure 12**).

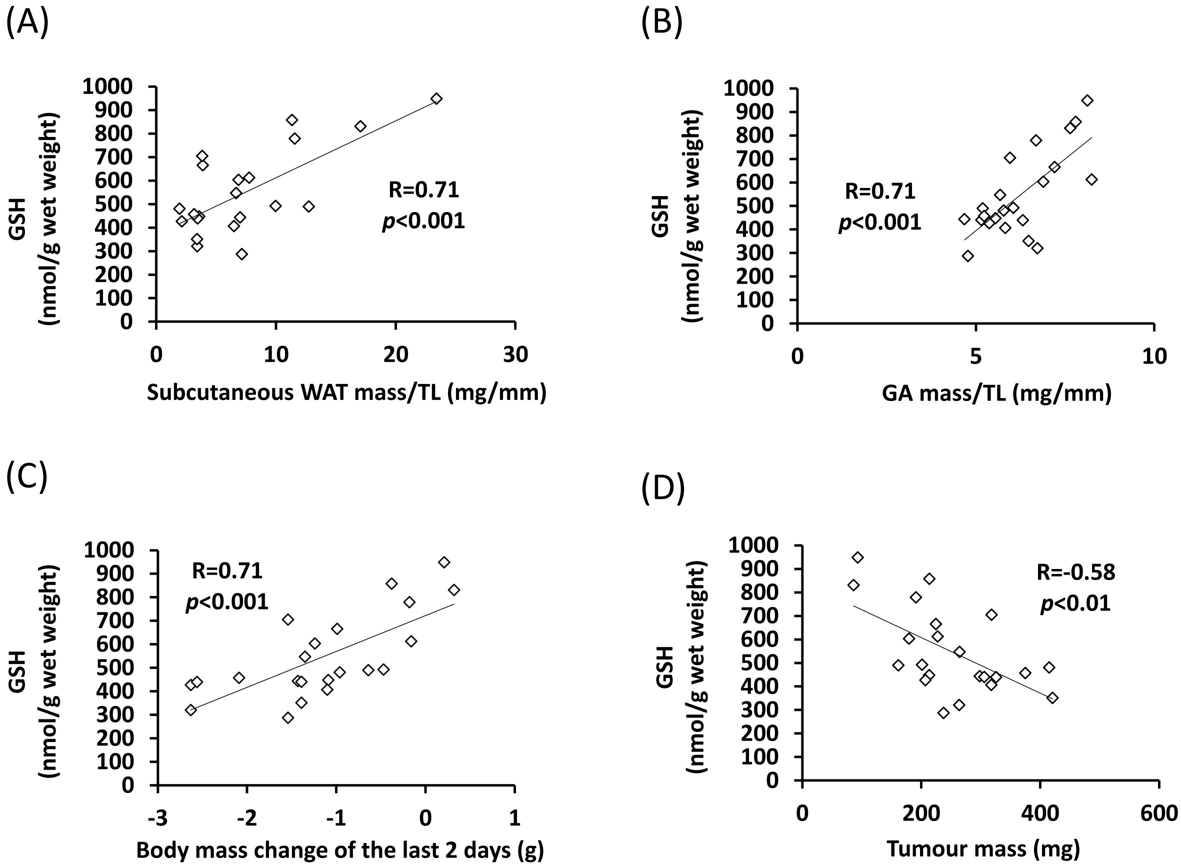

Supplementary figure 2. Correlation between skeletal muscle reduced glutathione (GSH) and (A) subcutaneous white adipose tissue (WAT) mass per the length of tibia, (TL) (B) gastrocnemius (GA) mass per TL, (C) body mass change of the last 2 days of the experiment and (D) tumor mass. All C26 tumor-bearing mice were included for the correlation analysis (*n* = 22).

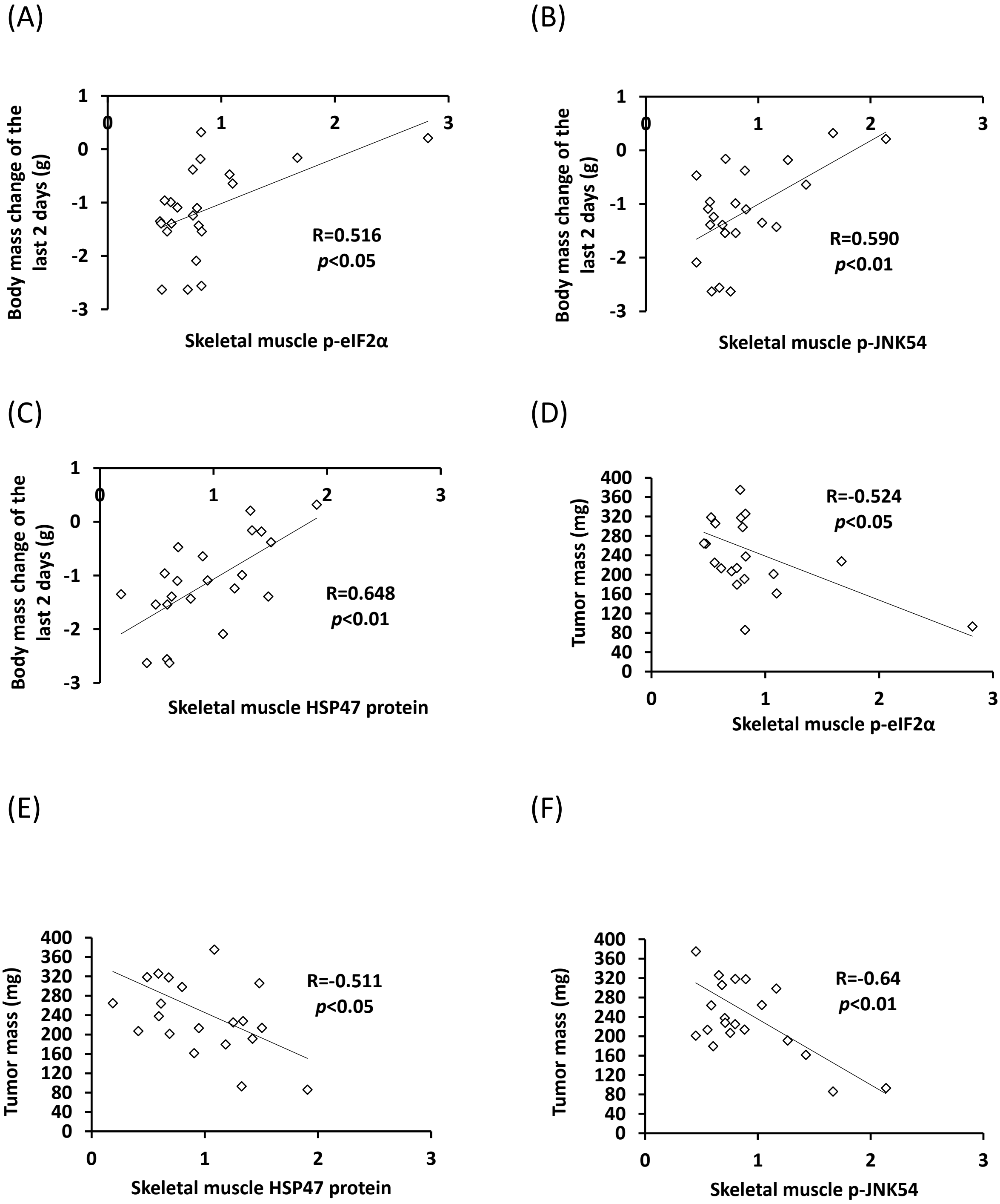

Supplementary figure 3. Correlation between the body mass change of the last 2 days of the experiment and skeletal muscle (A) p-eIF2α, (B) HSP47 and (C) p-JNK54. Correlation between the tumor mass and skeletal muscle (D) p-eIF2α^{ser51}, (E) HSP47 and (F) p-JNK54. All C26 tumor-bearing mice were included for the correlation analysis (n = 22).

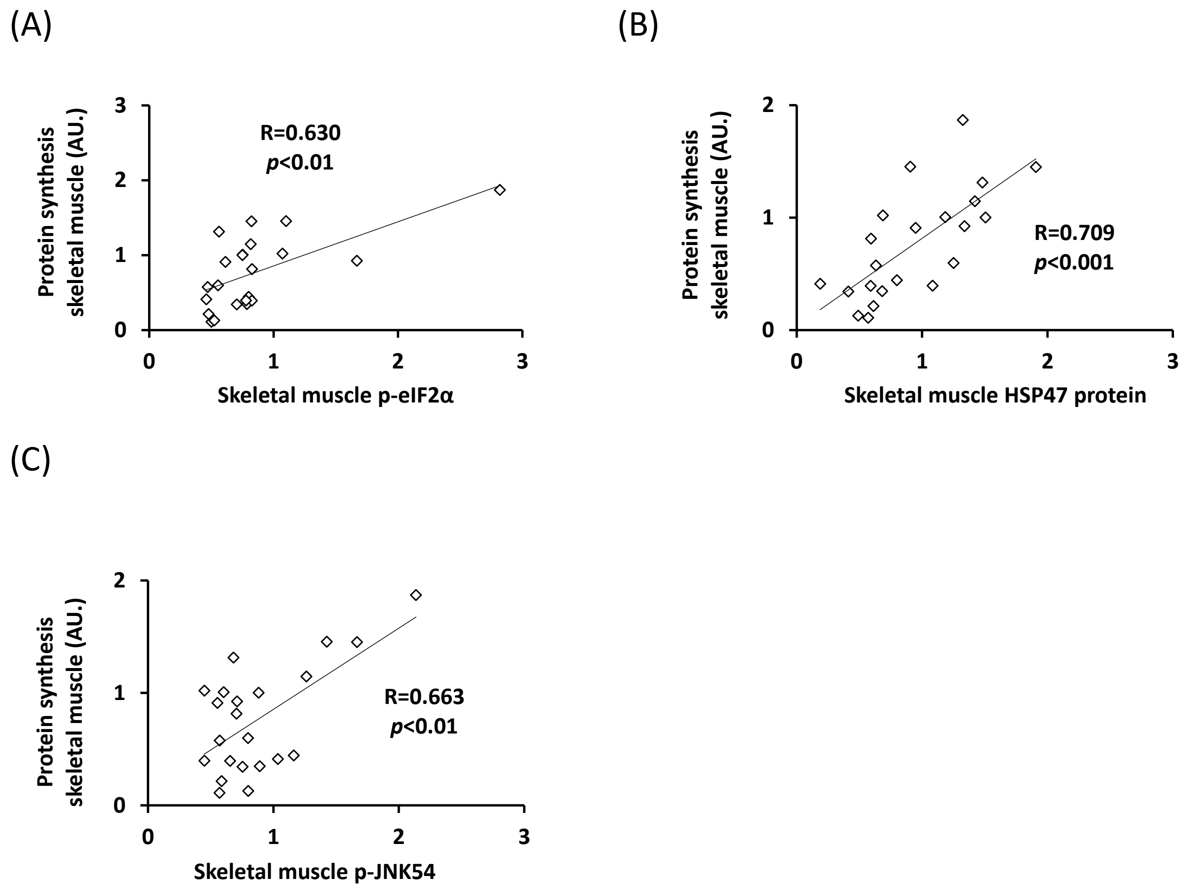

Supplementary figure 4. Correlation between skeletal muscle protein synthesis (A.U.) and skeletal muscle (A) p-eIF2α^{ser51}, (B) HSP47 and (C) p-JNK54. All C26 tumor bearing mice were included for the correlation analysis (*n* = 22). Protein synthesis was measured with SunSET method as previously described (Nissinen et al., 2018).

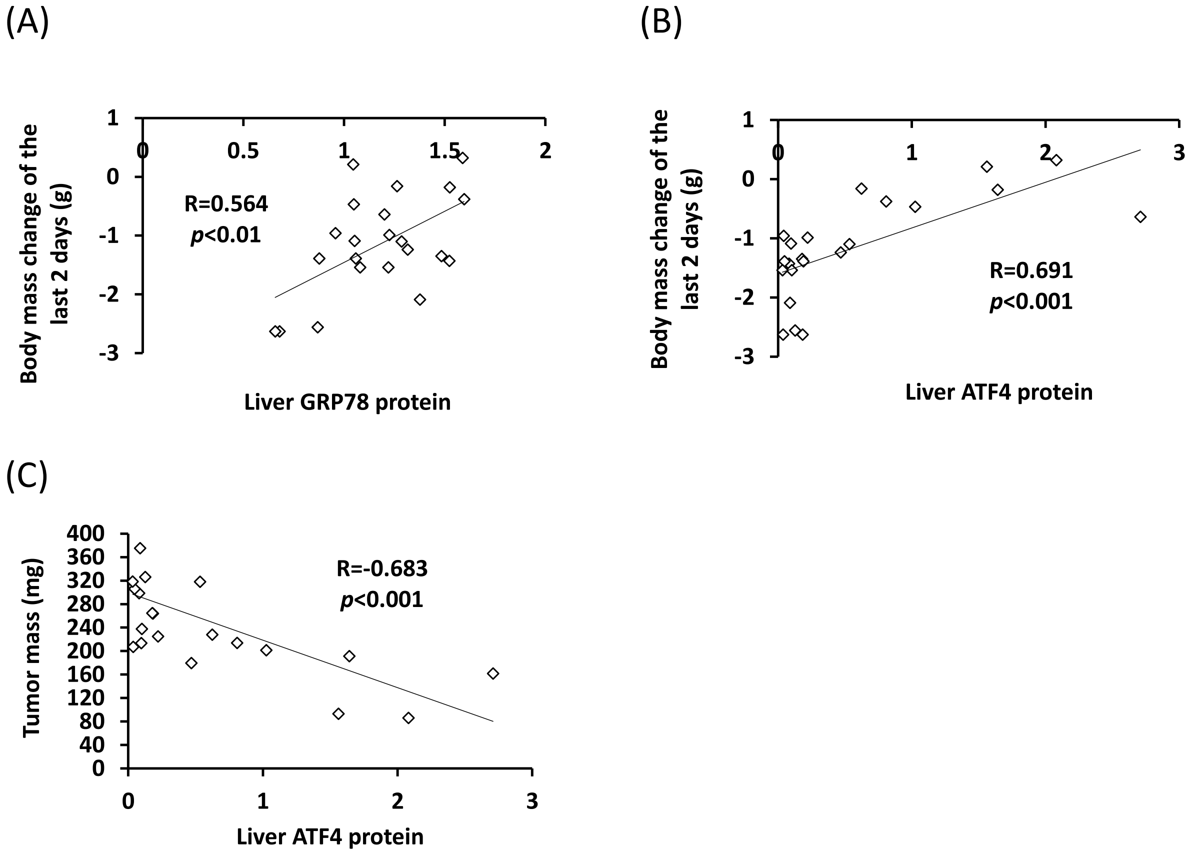

Supplementary figure 5. Correlation between (A) the body mass change of the last 2 days of the experiment and liver GRP78 protein, (B) the body mass change of the last 2 days of the experiment and liver ATF4 protein and (C) correlation between the liver ATF4 protein and tumor mass. All C26 tumor-bearing mice were included for the correlation analysis (*n* = 22).

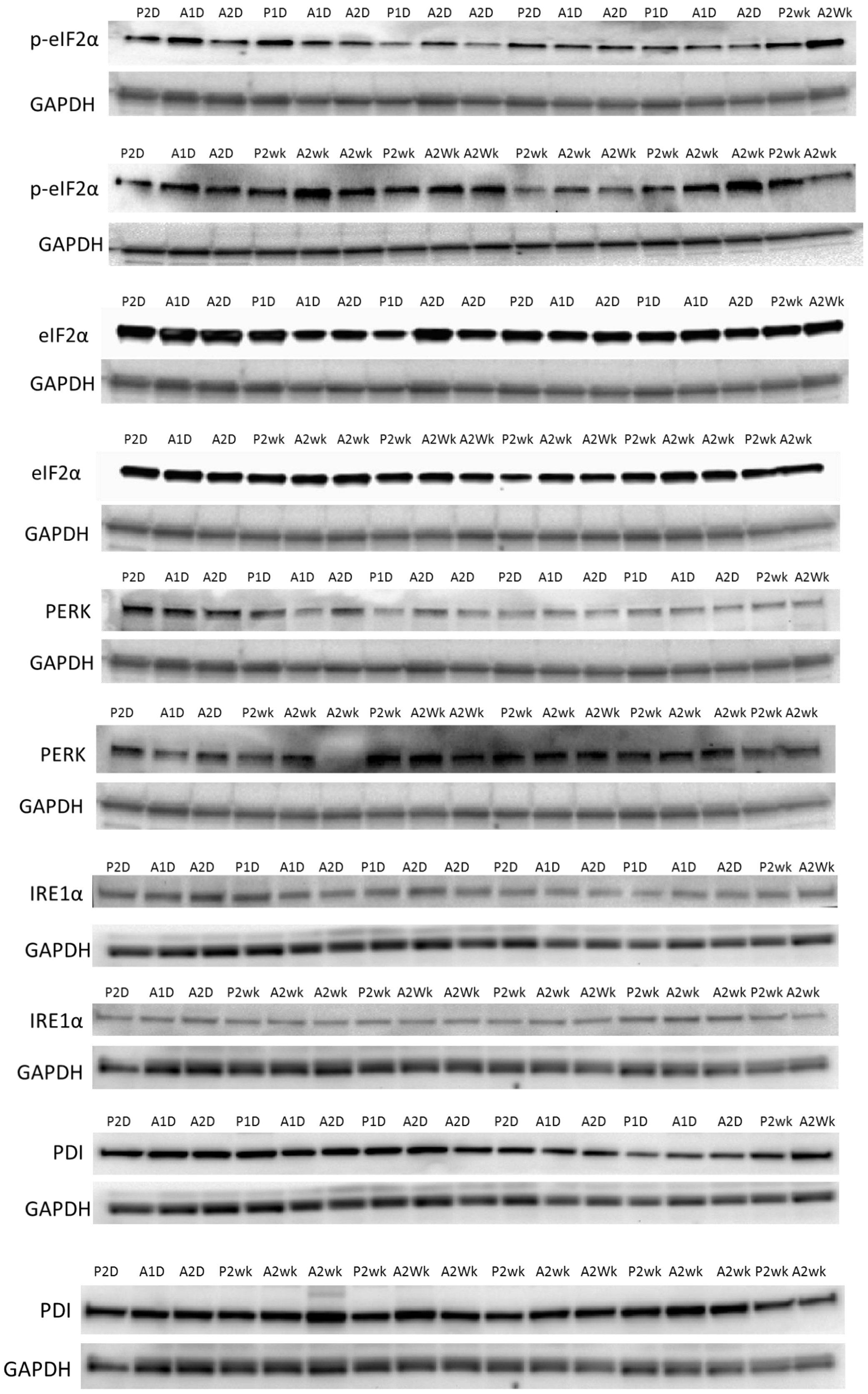

Supplementary figure 6. Original western blot images of the acute 1 – 2 d experiment and 2 week experiment. P = PBS, A = sACVR , 1D = muscles collected 1 day after a single sACVR or PBS administration, 2D = muscles collected 2 days after a single sACVR or PBS administration, 2Wk = muscles collected after 2 weeks of sACVR or PBS administration.

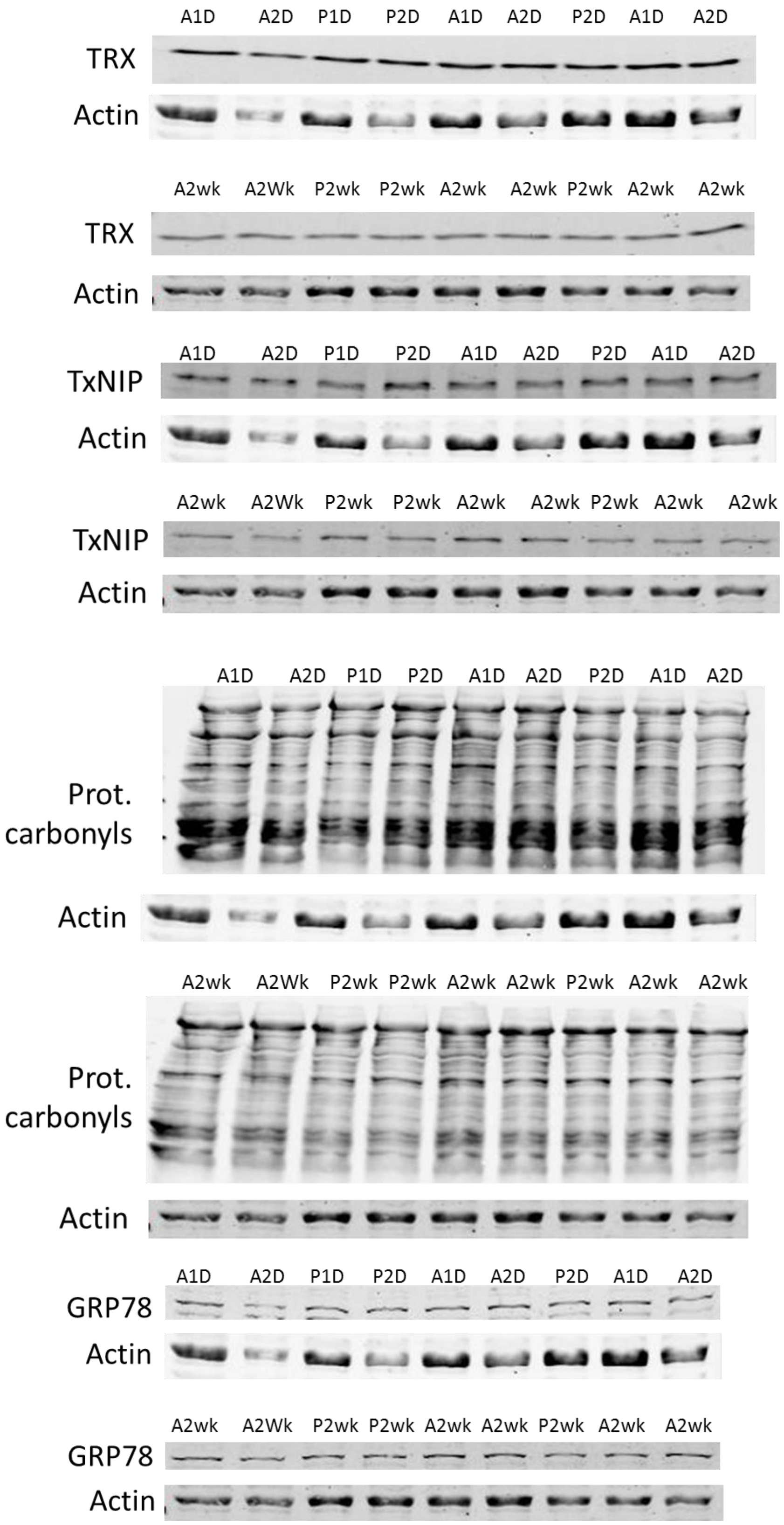

Supplementary figure 7. Original western blot images of the acute 1 – 2 d experiment and 2 week experiment. P = PBS, A = sACVR, 1D = muscles collected 1 day after a single sACVR or PBS administration, 2D = muscles collected 2 days after a single sACVR or PBS administration, 2Wk = muscles collected after 2 weeks of sACVR or PBS administration.

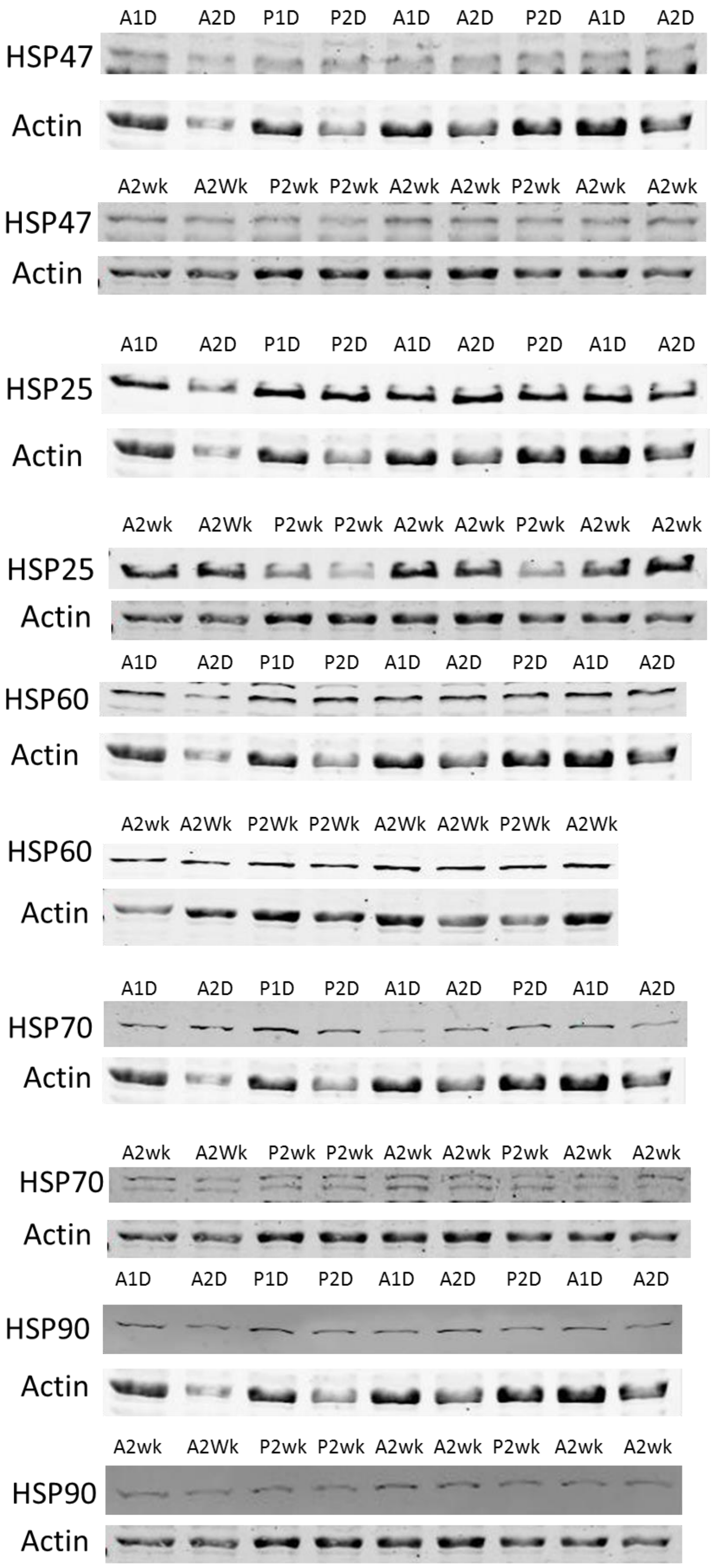

Supplementary figure 8. Original western blot images of the acute 1 – 2 d experiment and 2 week experiment. P = PBS, A = sACVR, 1D = muscles collected 1 day after a single sACVR or PBS administration, 2D = muscles collected 2 days after a single sACVR or PBS administration, 2Wk = muscles collected after 2 weeks of sACVR or PBS administration.

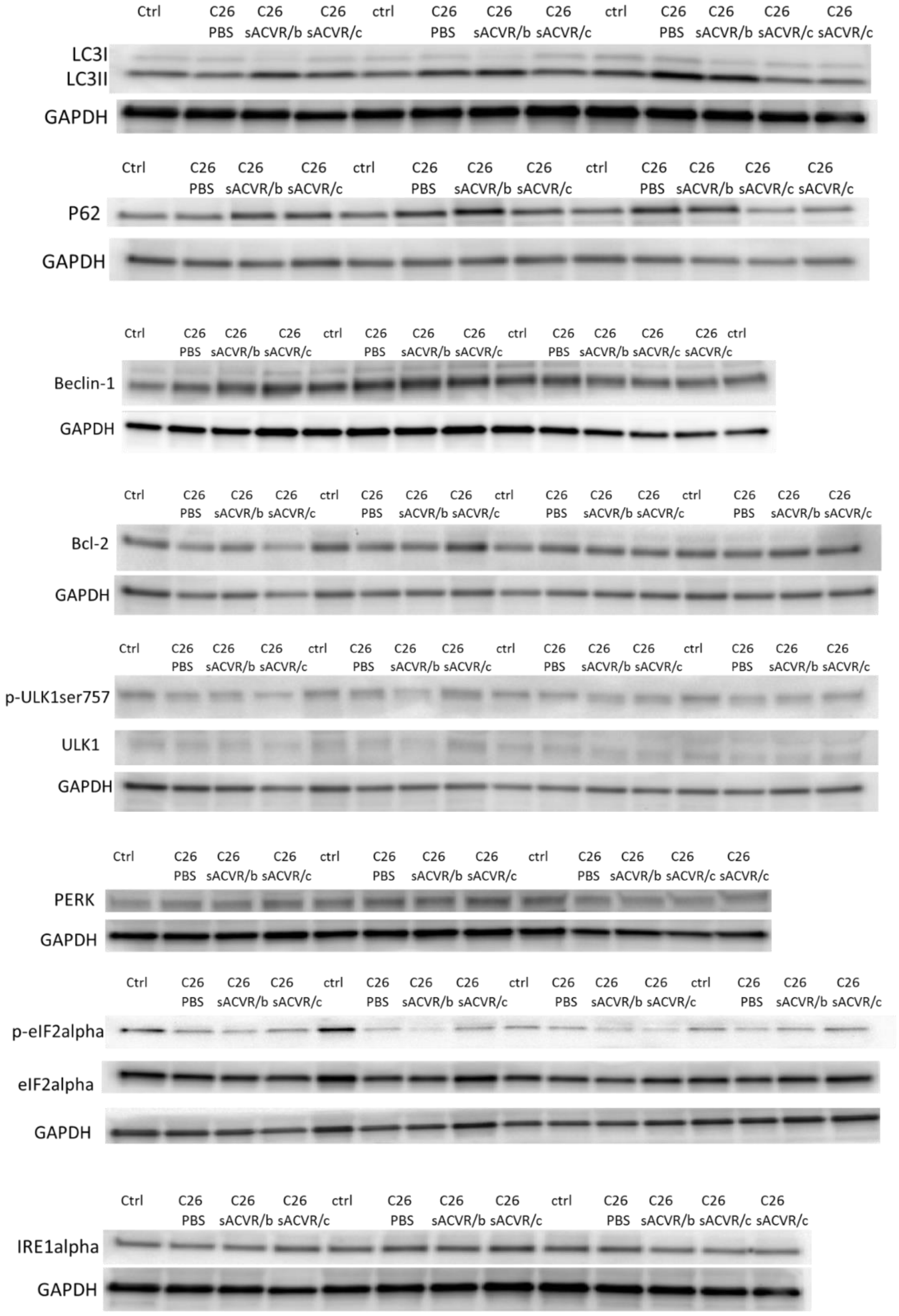

Supplementary figure 9. Original western blot images of the C26 experiment (skeletal muscle). CTRL = vehicle-treated (PBS) healthy control mice. C26 + PBS = C26 tumor-bearing mice administered with a vehicle (PBS), C26 + sACVR/b = C26 tumor-bearing mice administered with sACVR before the tumor formation and replaced by a vehicle (PBS) after the tumor formation, C26 + sACVR/c = C26 tumor-bearing mice continuously administered with sACVR2B-Fc throughout the experiment.

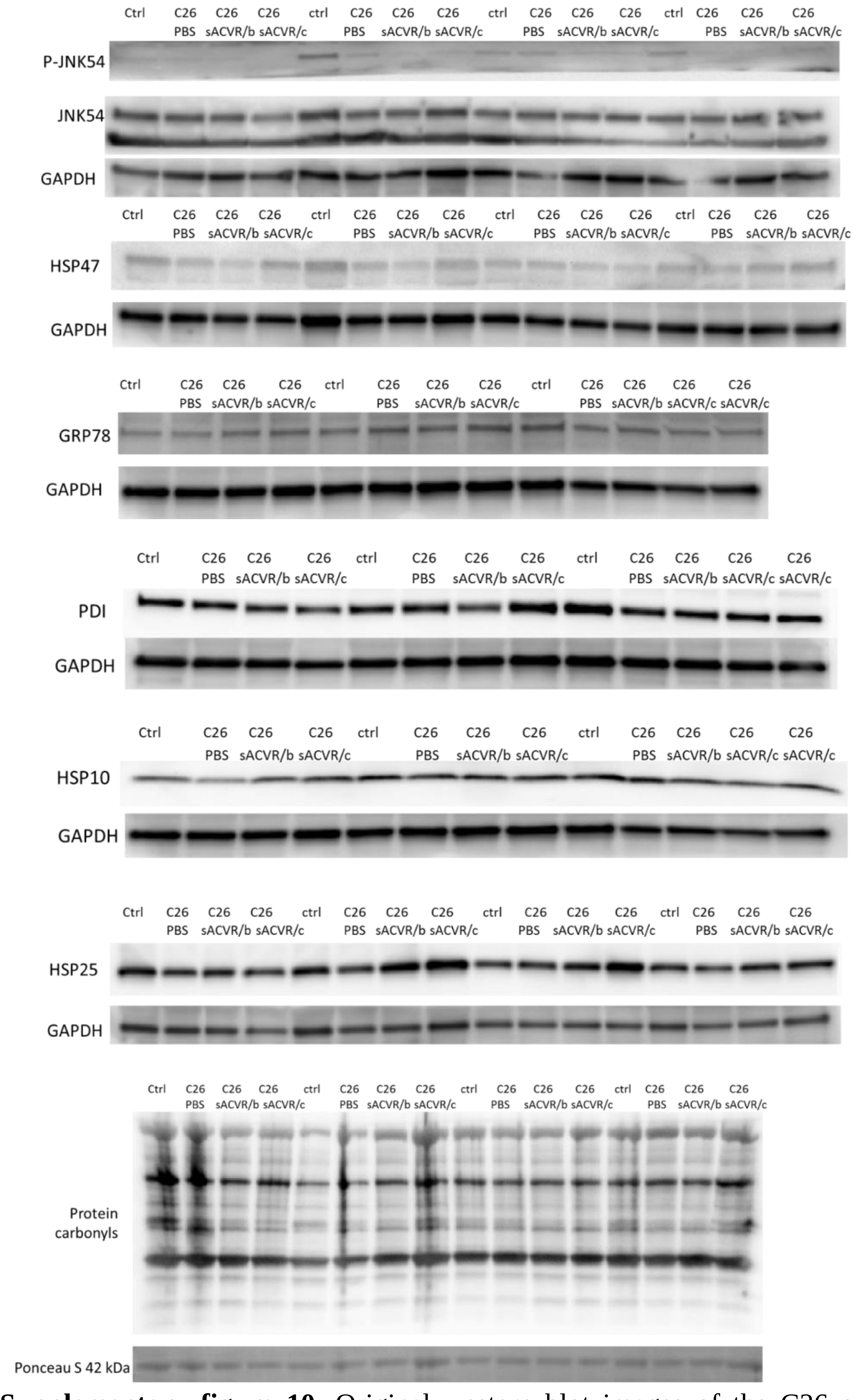

Supplementary figure 10. Original western blot images of the C26 experiment (skeletal muscle). CTRL = vehicle-treated (PBS) healthy control mice. C26 + PBS = C26 tumor-bearing mice administered with a vehicle (PBS), C26 + sACVR/b = C26 tumor-bearing mice administered with sACVR before the tumor formation and replaced by a vehicle (PBS) after the tumor formation, C26 + sACVR/c = C26 tumor-bearing mice continuously administered with sACVR2B-Fc throughout the experiment.

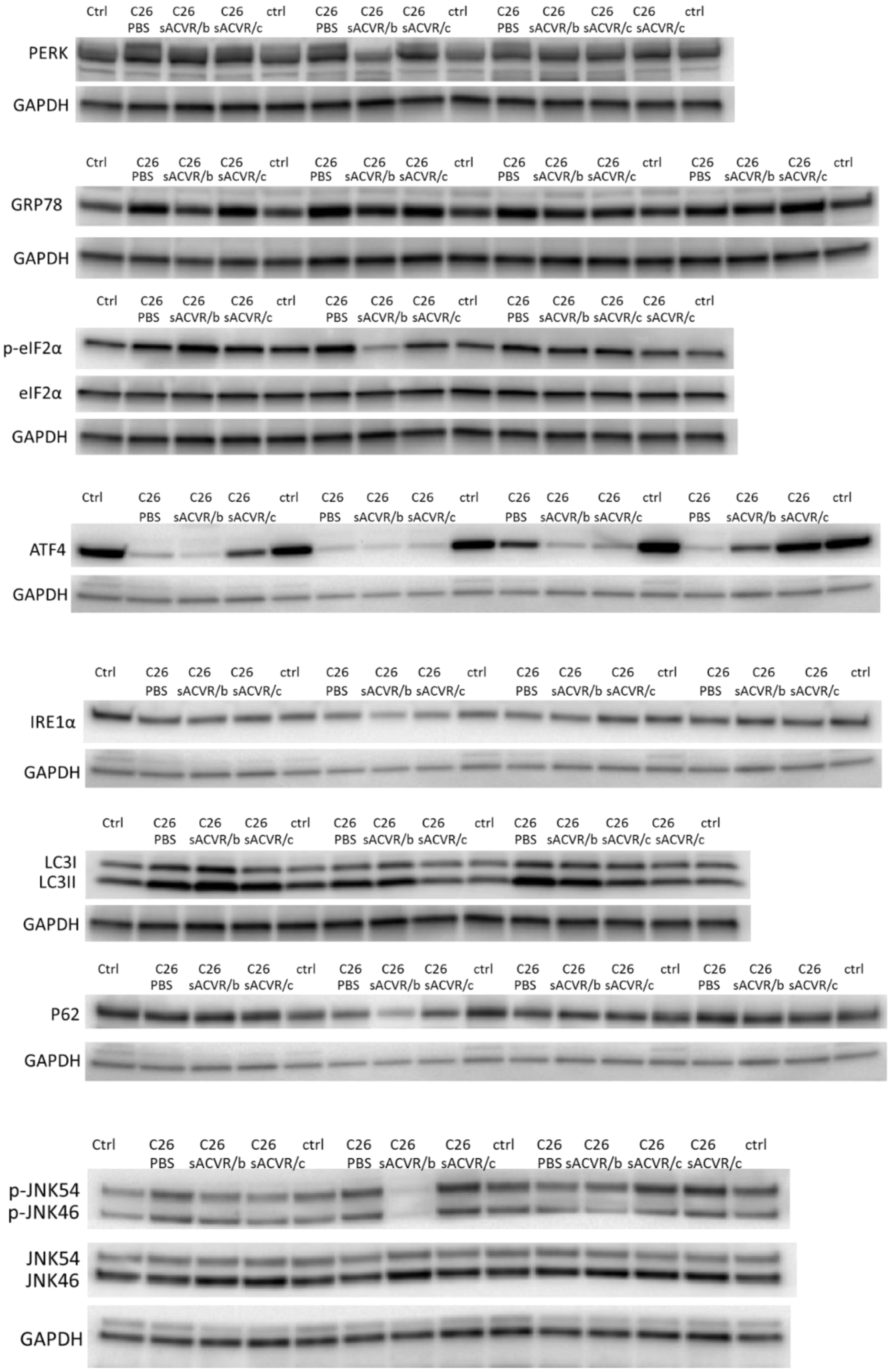

Supplementary figure 11. Original western blot images of the C26 experiment (liver). CTRL = vehicle-treated (PBS) healthy control mice. C26 + PBS = C26 tumor-bearing mice administered with a vehicle (PBS), C26 + sACVR/b = C26 tumor-bearing mice administered with sACVR before the tumor formation and replaced by a vehicle (PBS) after the tumor formation, C26 + sACVR/c = C26 tumor-bearing mice continuously administered with sACVR2B-Fc throughout the experiment.

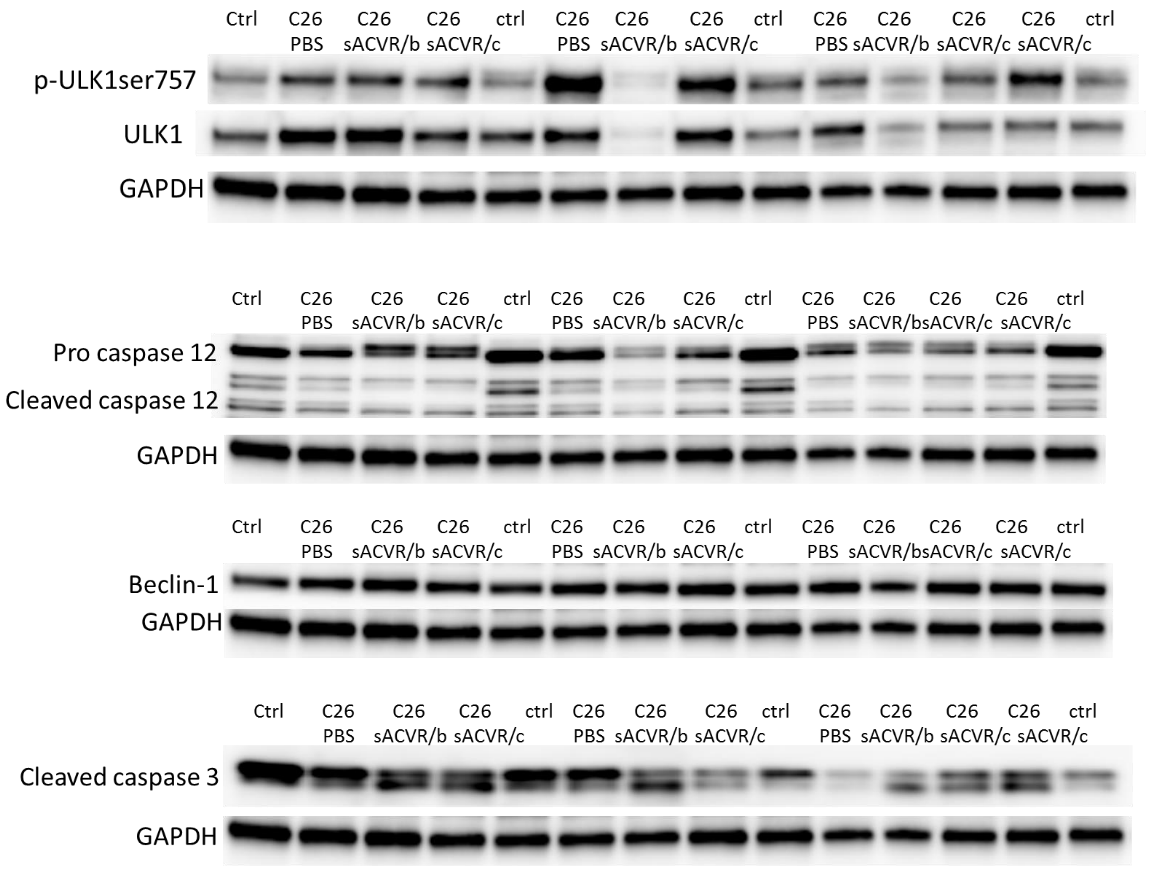

Supplementary figure 12. Original western blot images of the C26 experiment (liver). CTRL = vehicle-treated (PBS) healthy control mice. C26 + PBS = C26 tumor-bearing mice administered with a vehicle (PBS), C26 + sACVR/b = C26 tumor-bearing mice administered with sACVR before the tumor formation and replaced by a vehicle (PBS) after the tumor formation, C26 + sACVR/c = C26 tumor-bearing mice continuously administered with sACVR2B-Fc throughout the experiment.
